# Imaging Biomarkers and Pathobiological Profiling in a Rat Model of Drug-Induced Interstitial Lung Disease Induced by Bleomycin

**DOI:** 10.3389/fphys.2020.00584

**Published:** 2020-06-19

**Authors:** Irma Mahmutovic Persson, Hanna Falk Håkansson, Anders Örbom, Jian Liu, Karin von Wachenfeldt, Lars E. Olsson

**Affiliations:** ^1^Department of Medical Radiation Physics, Institution of Translational Medicine, Faculty of Medicine, Lund University, Malmö, Sweden; ^2^Truly Labs, Lund, Sweden; ^3^Division of Oncology and Pathology, Department of Clinical Sciences, Lund University, Lund, Sweden; ^4^TRISTAN-IMI Consortium (Translational Imaging in Drug Safety Assessment-Innovative Medicines Initiative)

**Keywords:** drug-induced interstitial lung disease, imaging, animal model, bleomycin, lung injury, magnetic resonance imaging, positron emission tomography, inflammation

## Abstract

A large number of systemically administered drugs have the potential to cause drug-induced interstitial lung disease (DIILD). We aim to characterize a model of DIILD in the rat and develop imaging biomarkers (IBs) for detection and quantification of DIILD. In this study, Sprague–Dawley rats received one single dose of intratracheal (i.t.) bleomycin and were longitudinally imaged at day 0, 3, 7, 14, 21, and 28 post dosing, applying the imaging techniques magnetic resonance imaging (MRI) and positron emission tomography (PET)/computed tomography (CT). Bronchoalveolar lavage fluid (BALF) was analyzed for total protein and inflammatory cells. Lungs were saved for further evaluation by gene analysis using quantitative-PCR and by histology. Lung sections were stained with Masson’s-Trichrome staining and evaluated by modified Ashcroft score. Gene expression profiling of inflammatory and fibrotic markers was performed on lung tissue homogenates. Bleomycin induced significant increase in total protein concentration and total cell count in bronchoalveolar lavage (BAL), peaking at day 3 (*p* > 0.001) and day 7 (*p* > 0.001) compared to control, respectively. Lesions measured by MRI and PET signal in the lungs of bleomycin challenged rats were significantly increased during days 3–14, peaking at day 7. Two subgroups of animals were identified as low- and high-responders by their different change in total lung volume. Both groups showed signs of inflammation initially, while at later time points, the low-responder group recovered toward control, and the high-responder group showed sustained lung volume increase, and significant increase of lesion volume (*p* < 0.001) compared to control. Lastly, important inflammatory and pro-fibrotic markers were assessed from lung tissue, linking observed imaging pathological changes to gene expression patterns. In conclusion, bleomycin-induced lung injury is an adequate animal model for DIILD studies and for translational lung injury assessment by MRI and PET imaging. The scenario comprised disease responses, with different fractions of inflammation and fibrosis. Thereby, this study improved the understanding of imaging and biological biomarkers in DIILD and lung injury.

## Introduction

Many drugs generate more or less severe adverse effects, affecting one or several organs depending on the type of drug, dosing, and administrative route. When adverse effects afflict the lung upon drug administration, the interstitium is harmed inducing DIILD, a disease that in the worst case can progress into lung failure, chronic fibrosis, and death (Flieder and Travis, 2004; Schwaiblmair et al., 2012; Delaunay et al., 2017). The incidence of DIILD could be expected to increase since there is an increasing number of new drugs and therapies developed as well as an increasing global aging population (Matsuno, 2012).

Drug-induced interstitial lung disease is a form of ILD, caused by drugs and not associated with environmentally induced damage caused by inhaled agent(s) (Travis et al., 2013; Antoniou et al., 2014). Despite a clear classification of DIILD, it is difficult to detect and distinguish it from other ILD conditions. Different drugs can induce very different pathophysiologies, and other already present pathological conditions in the lung may hamper the diagnosis of DIILD (Camus et al., 2004; Skeoch et al., 2018). The disease is generally only suspected when radiological assessment indicates an onset of ILD upon drug administration and when other causes of ILD have been ruled out. However, there is not one single characteristic pathophysiological or specific radiological pattern for how this disease manifests itself, but rather a number of different appearances in which it can be manifested (Travis et al., 2013; Skeoch et al., 2018).

A simplified description of the time course of DIILD might involve plasma exudation giving rise to edema and inflammation in the lung during the early phase, while later stages include fibrosis (Schwaiblmair et al., 2012; Skeoch et al., 2018). The fibrotic progression is initiated upon activation of pro-fibrotic pathways including production of TGF-β and CTGF, and subsequent deposition of collagen and other ECM proteins (Thannickal et al., 2004; Cox and Erler, 2011; Wynn, 2011; Wynn and Ramalingam, 2012). Activation and differentiation of cells that mediate and contribute to the fibrotic milieu in the lung induce formation of fibrotic loci that may further evolve into established fibrotic scaring of the lung parenchyma. Together these events contribute to lung tissue damage and eventually result in aggravated breathing difficulties (Matsuno, 2012; Schwaiblmair et al., 2012; Delaunay et al., 2017). Once DIILD is diagnosed, drug withdrawal and corticosteroid treatment can be initiated (Camus et al., 2004; Skeoch et al., 2018). The time for diagnosis and treatment is crucial in order to reduce morbidity and mortality in patients with DIILD. Therefore, in order to enable early detection of disease progression, better tools are warranted for early diagnosis and timely management.

Despite the fact that several hundreds of drugs are able to cause DIILD, there are no efficient biomarkers to date for diagnostic or prognostic purposes (Biomarkers Definitions Working Group, 2001; Sullivan et al., 2015; FNB Working Group, 2016; Pneumotox, 2020; Tristan, 2020). To develop biomarkers that are non-invasive, yet able to detect disease onset and progression as early as possible, IBs have been suggested as a prominent tool. IBs would offer good translatability between clinical and preclinical research besides optimal possibilities to monitor disease progression but also resolution (Barreto et al., 2013; Skeoch et al., 2018). Thus, in order to develop these IBs as well as work on the translational properties, well-characterized preclinical DIILD models are needed. In this study, IBs are studied based on MRI and PET.

In patients with suspected DIILD, imaging is already part of the diagnosis where HRCT imaging is the current method of choice (Padley et al., 1992; Potente et al., 1992). However, in addition to HRCT, PET has been applied using FDG coupled to ^18^F (FDG-PET), to provide useful information of early signs of DIILD (Paschali et al., 2017). The use of FDG-PET could be of particular interest in indications where it is already being used to diagnose or follow the disease for which the DIILD-inducing drug is prescribed, since here baseline data would be available. One such example is Hodgkin’s disease where treatment with doxorubicin, bleomycin, vinblastine, and dacarbazine (ABVD) is considered standard of care (Paschali et al., 2017). Bleomycin is one of the drugs with highest incidence of DIILD occurring in 8–10% of patients treated with bleomycin (Matsuno, 2012; Schwaiblmair et al., 2012; Skeoch et al., 2018).

In order to develop relevant animal models that can mimic DIILD, various phases of the disease should optimally be present in such a model. Bleomycin is most often used as an inducing agent in preclinical fibrosis models. It is a typical agent that can induce initial inflammation followed by progressive fibrosis that in many ways resembles the clinical manifestations of DIILD (Izbicki et al., 2002; Bethany et al., 2013). However, in these studies, the bleomycin lung instillation model is mostly studied in its later stages due to its resemblance to IPF (Moeller et al., 2008). The classical bleomycin model is currently considered as the most appropriate model to use due to its robust repeatability and reproducibility (Bonniaud et al., 2018). Therefore, we initiated our development of IBs for DIILD within this model.

The aim of this study was to characterize a model of DIILD using i.t. bleomycin challenge in the rat and investigate how MRI and PET can be used to monitor the progression of DIILD. The main objective was to identify IBs that can distinguish different pathological changes such as inflammation and fibrosis in the bleomycin-induced DIILD model. Both *ex vivo* analyses of tissue, cells and gene expressions were analyzed. Pathobiological changes in bronchoalveolar lavage (BAL) and lung tissue were related to data obtained by MRI and FDG-PET.

## Materials and Methods

### Animals

In total, 45 Sprague–Dawley male rats from Taconic (Taconic, Lille Skensved, Denmark) were used in these studies. The rats were 7–10 weeks of age (250–400 g) and were housed at Lund University and Medicon Village animal facilities with 12 h light/dark cycles and fed *ad libitum*. Before the start of the study, the experimental procedures were evaluated and approved by the local ethical committee in Lund/Malmö, Sweden, with permit numbers 4003/2017 and 3226/2017. In addition, all animal studies were ethically reviewed and carried out in accordance with European Directive 2010/63/EEC and ARRIVE guidelines (Kilkenny et al., 2010).

**FIGURE 1:**
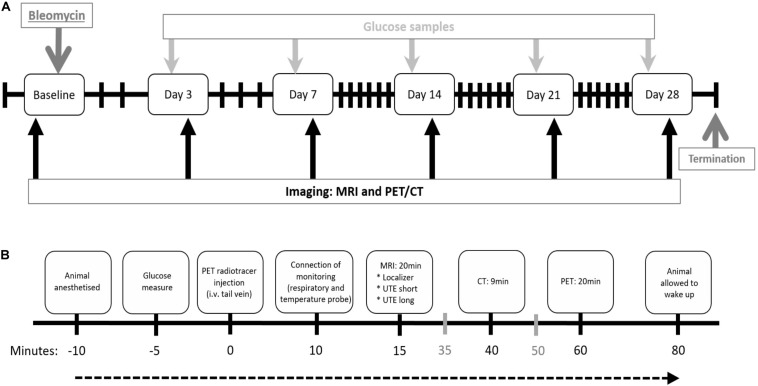
Study layout and imaging workflow chart. **(A)** Study layout of the DIILD model with bleomycin-induced lung injury in Sprague–Dawley male rats. Imaging was performed at baseline (scan 1) before the intratracheal (i.t.) administration of bleomycin. Control rats received saline as i.t. challenge. Imaging sessions occurred at day 0, 3, 7, 14, 21, and 28 post challenge. Before every scan session, blood glucose was measured. Termination occurred at day 28, after the final imaging session. **(B)** Workflow for each imaging session. Events expressed in gray (35 and 50 min after injection) indicate when the following rat is prepared while the scan of the previous rat is being performed. i.v. = intravenous.

At arrival, the rats underwent a health check and were allowed to acclimatize to the housing conditions for a minimum of 5 days before any experimental procedures. Animals were divided in two main groups, the imaging group (*n* = 12) and non-imaging group (*n* = 33). Saline challenged rats were present in both groups as controls. Imaging sessions or termination of animals were performed at day 0, 3, 7, 14, 21, and day 28 post bleomycin challenge (Figure 1A). The rats in the imaging group were scanned longitudinally (Figure 1B) while three to five rats from the non-imaging group were terminated at each time point for sampling of BALF and collection of lung tissue.

Power calculations and additional animal handling are described in Supplementary Data File S1.

### Bleomycin Challenge

The rats received a single i.t. dose of bleomycin (Sigma–Aldrich, St. Louis, MO, United States), with a concentration of 1000 iU in 200 μl saline. Control animals received the same volume of saline only. The procedure of i.t. administration was performed by placing the lightly anesthetized (IsoFlo^®^ vet, Orion Pharma, Sweden) rat, in a supine position on a slanting board. The i.t. administration was performed with a syringe connected to a blunt cannula with a small steel marble at the top. The administration of bleomycin or saline solution was performed after the cannula passed the larynx and the rings of cartilage in the trachea could be felt. The 200 μl of bleomycin or saline solution was followed by an immediate puff of air, for optimal distribution of the solution.

### *In vivo* Imaging

Imaging was performed using MRI and PET/CT at the LBIC. The different imaging modalities were combined consecutively in one workflow, for each animal and imaging session, as described thoroughly in Figure 1B, and shown in Supplementary Figure S1. In order to facilitate the co-registration of the MRI and PET/CT images, the animal was kept in the same animal bed throughout the session. To achieve this, the animal bed from the PET/CT scanner (Minerve imaging cells, Minerve, France) was used, and an adapter was designed and printed in-house, using a 3D-printer (Ultimaker 2, Geldermalsen, Netherlands). This adapter facilitated the connection between the Bruker animal positioning system and the Minerve animal holder providing warm air, anesthesia, and temperature/respiration monitoring.

The rats were anesthetized using isoflurane delivered via a nose cone and the PET radiotracer ^18^F-FDG was injected intravenously via the tail vein, allowing the tracer to circulate systemically for approximately 1 h before PET imaging. In the meantime, the rat was placed in the animal bed and imaged on the MRI system. Immediately after MRI, PET/CT imaging was performed. The CT images were obtained for anatomical orientation. All imaging sessions were performed on spontaneously breathing animals without any heart or respiratory triggering. The respiratory rate was monitored using a pneumatic pillow and regulated by adjusting the flow of isoflurane. Approximately 1–2% isoflurane was used during imaging sessions. Respiratory rate was kept to 70–90 breaths/min. Similarly, the body temperature, monitored by a rectal probe, was kept constant by adjusting the temperature of the heating pad in the animal bed.

Prior to animal scan, body weight was noted and a blood glucose test was performed. Blood was drawn from the paw and the glucose level analyzed using a Glucose 201 RT (HemoCue, Sweden).

### MRI

Rats were imaged on a preclinical 9.4T MRI Biospec AV III (Bruker, Ettlingen, Germany) using ParaVision 6.0.1 (Bruker). The rats were initially scanned applying a low resolution scan (Localizer) to confirm optimal positioning of the animal in the scan. Thereafter two different UTE sequences were applied using echo time (TE) referred to as short (TE_SHORT_) with 0.324 ms or long (TE_LONG_) with 1 ms. TR was 8 ms (depending on the number of slices), and the flip angle was 25°. The FOV was 58 × 58 mm^2^ (matrix size 192 × 192) and the 2D transverse slices were 2 mm in thickness. Images were reconstructed and zero filled to a 256 × 256 matrix and a reconstructed pixel resolution of 0.22 × 0.22 mm^2^. For the imaging, an 86 mm diameter quadrature body RF coil was used (Bruker, Ettlingen, Germany). Depending on the size of the animal, a total of 19 ± 3 slices were used to cover the whole lung. As the acquisition was not triggered on the respiratory or cardiac motion, a total of four averages were used to reduce motion artifacts. The experimental time was 25 ± 5 min in total for MRI, including all preparations; from positioning the animal into the animal bed, connecting the various sensors (breathing, temperature) and anesthesia, through the various TE scans and repositioning of the FOV.

### PET/CT

Immediately after the MRI scan session, the rat in the animal bed was transferred and connected to the PET/CT system; (nanoScan^®^ PET/CT, Mediso, Hungary) for CT and PET imaging. Initially a fast CT scan (scout-view) was performed with 11 s acquisition, to adjust the position of the animal to cover the lungs. CT scan, used for localization and attenuation correction, was performed at a total scan time of 9 min including post-reconstruction. The CT scan parameters used were: 65 kVp, an exposure time of 500 ms, and a voxel size of 0.14 × 0.14 × 0.14 mm^3^. Immediately after CT imaging, a PET scan was performed. The PET radiotracer had been allowed to circulate systemically during 1 h (±5 min) until PET measurements were initiated. The radiotracer ^18^F-FDG was injected at a dose of 30 (±5) MBq diluted in a volume of 200 μl saline. Production of ^18^F-FDG was done at Section of Nuclear Medicine (Radiation Physics, Skåne University Hospital, Sweden), providing radioligands for medical PET imaging. The radiotracer was delivered after quality control tests to LBIC, where imaging was performed. The PET scan was acquired using a scan time of 20 min. Post-reconstruction of PET data was performed using the instrument protocol of MLEM with four iterations and six subsets (Nucline^TM^, Mediso, Hungary). Voxel size after reconstruction was 0.4 × 0.4 × 0.4 mm^3^.

### Imaging Data Analysis

All imaging data were saved as DICOM files. CT and PET images were post-reconstructed and stored on the same drive as the MRI raw data. All images were then reviewed qualitatively and quantified using the software VivoQuantTM 3.5 (inviCRO Imaging Services and Software VivoQuant^1^). The imaging quantification workflow was initiated by drawing ROIs in each MRI slice section, throughout the whole lung, in each rat being scanned. The ROI in each slice was drawn manually guided by template manuals (using the “Spline tool”) within the software. The ROI did not include the heart or vessels with clear attachment to the heart. Thorough demonstration of ROI selection of various slices, within one animal scan, is given in Supplementary Figure S2.

Once each ROI was completed within one animal scan, histograms of the data contained in the ROI were exported for both TE_SHORT_ and TE_LONG_. The MRI histograms were batch analyzed using custom software written for the purpose in IDL 8.5 (Exelis VIS, Harris Corporation, Boulder, CO, United States). The software identifies the first peak in each histogram, representing the low-signal voxels in the normal lung. The point on the slope at the right side of this peak with the sharpest decline is then found and a linear function tangential to the slope is calculated. The sum of the volume in the histogram with signal above where the linear function intersects with the *x*-axis was defined as the MRI “high-signal” volume for each histogram, as shown in Supplementary Figure S3A. The image that can be generated from the MRI by applying the histogram data shows ROI of the total lung (in red) in relation to the “high-signal” ROI (cyan), as demonstrated in Supplementary Figure S3B.

The MRI TE_LONG_ data as well as the ROI were resampled into isotropic voxels before the PET and CT scans of the same animal, at the same time point, were imported into the software session. To match these disparate datasets, MRI and ROI were used as reference while an automatic registration was performed of PET and CT datasets to the MRI datasets. The settings of Rigid translation, and Fine quality, was employed in VivoQuantTM 3.5 which uses a mutual information metric to fit the datasets together using translations and rotations during a maximum of 200 iterations. Post registration, the activity distribution in the PET image within the lung ROI was measured, both as activity up-take per mm^3^ and total up-take in the whole lung, and divided by the decay-corrected injected dose per animal to find the fractional up-take.

### Termination of Experiment and Sample Collection

Rats from the imaging group were imaged longitudinally, with termination at the last day of the study (day 28) after the final imaging session was completed. Rats from the non-imaging groups were terminated continuously during the experiment, at day 3, 7, 14, 21, and 28 post bleomycin administration. At termination, the rats received an intraperitoneal overdose of Pentobarbital Sodium (Apotek Produktion and Laboratorier AB, Sweden). Subsequently, BAL was performed by letting PBS flow into the lungs with a pressure of 10 cm H_2_O for 2 min and collecting the lavage fluid. This procedure was repeated, resulting in two rounds of BAL. After BAL, the left lung was ligated and insufflated with paraformaldehyde 4% and the remaining lobes of the right lung were dissected out, weighed and snap frozen in −80°C until use.

### *Ex vivo* Tissue Sample Analysis

Collected BALF was immediately put on ice and further processed. BALF was spun down on a centrifuge (1000 *g*) for 10 min, at 4°C. The supernatant was used for protein determination (Bradford Protein Assay kit, Bio-Rad). The cell pellet was re-suspended in PBS and counted. Thereafter, the cell suspensions were diluted to optimal cell concentration for cytospin, the cells spun onto microscopic glass slides, stained with May-Grünwald and Giemsa stain, and a differential count performed. A minimum of 400 cells were counted from each sample.

### Histological Analysis

Left lung was fixed in 4% paraformaldehyde and subsequently paraffin embedded and sectioned into 4 μm thick sections. The whole left lung was sectioned in the sagittal plane at four various positions, to evaluate the spread of lesions and how homogenous the disease spread was within the whole left lobe. The sections were stained with Masson’s-Trichrome staining (Masson’s-Trichrome Stain Kit, Polysciences, Hirschberg an der Bergstrasse, Germany), to observe fibrotic lesions and collagen accumulation in the fibrotic foci. The sections were evaluated qualitatively and quantitatively by assessment of fibrotic score according to the modified Ashcroft scoring scale (Hubner et al., 2008). Four sections were evaluated in each animal and all sections were scored and subsequently presented as a quantitative score graph as well as representative images that were chosen for selected time points.

### Gene Expression Analysis From Lung Tissue Homogenates

Frozen lung tissue was used for gene expression analysis. First, the lung tissue was homogenized using gentleMACS^TM^ Dissociator (Miltenyi Biotec) with lysis buffer supplied within the RNA extraction kit RNeasy Mini kit (Qiagen) including β-mercaptoethanol (Sigma). RNA was extracted, concentration measured using a nanophotometer p330 (IMPLEN, Inc., California) and 2 μg of total RNA was reverse transcribed into cDNA, thereafter the fibrosis panel kit was used according to manufacturer’s recommendations to analyze genes of interest. Both the reverse transcription-kit (RT^2^ Profiler PCR Array) and the Rat Fibrosis q-PCR panel (PARN-120ZA) were obtained from Qiagen. In brief, cDNA samples and MasterMix were mixed in pre-coated plates provided by Qiagen, standard thermocycling was performed on a BioRad (CFX96^TM^ Real-Time System, C1000^TM^ Thermal Cycler) detection system and the PCR products were assessed using the software CFX Manager^TM^ Software. For quantification of gene expression, the ^ΔΔ^CT-method (Livak and Schmittgen, 2001) was applied. Expression levels for genes of interest were calculated in relation to the geometric mean of five different reference genes; β-actin, β-2-macroglobulin, hypoxanthine phosphoribosyltransferase 1 (HPRT1), lactate dehydrogenase A (LDHa), and ribosomal protein lateral stalk subunit P1 (RPLP1). Gene expression was then normalized to its control sample at each time point. Quantified gene expression data was presented as mean ± standard error of the mean (SEM).

### Statistical Analysis

Data are expressed as mean values and SEM unless otherwise specified. All data were analyzed using non-parametric tests using the software GraphPad Prism (version 8.2 GraphPad Software, San Diego CA, United States^2^) and IBM SPSS (version 23, IBM, Somers, NY, United States). The one-way ANOVA test was applied to find differences between the groups, followed by *post hoc* analysis using Bonferroni’s multiple comparisons test. The two-tailed Mann–Whitney test was used to compare variance between groups. *P*-values of less than 0.05 were considered statistically significant. Significance is indicated as follows; when comparing bleomycin toward the saline control from the same time point significance is indicated by ^∗^ when *p* < 0.05; *p* < 0.01 by ^∗∗^; *p* < 0.001 by ^∗∗∗^, and *p* < 0.0001 by ^****^. The comparison of various time points between bleomycin-challenged groups is expressed as # when *p* < 0.05; ## when *p* < 0.01; ### when *p* < 0.001, and #### when *p* < 0.0001. When comparing toward baseline, within the same group of animals, significance is indicated by §when *p* < 0.01; *p* < 0.01 by §§, and *p* < 0.001 by §§§, as in the case of FDG-PET signal up-take.

## Results

### General Health Check and Measured Parameters During Imaging Sessions

**FIGURE 2:**
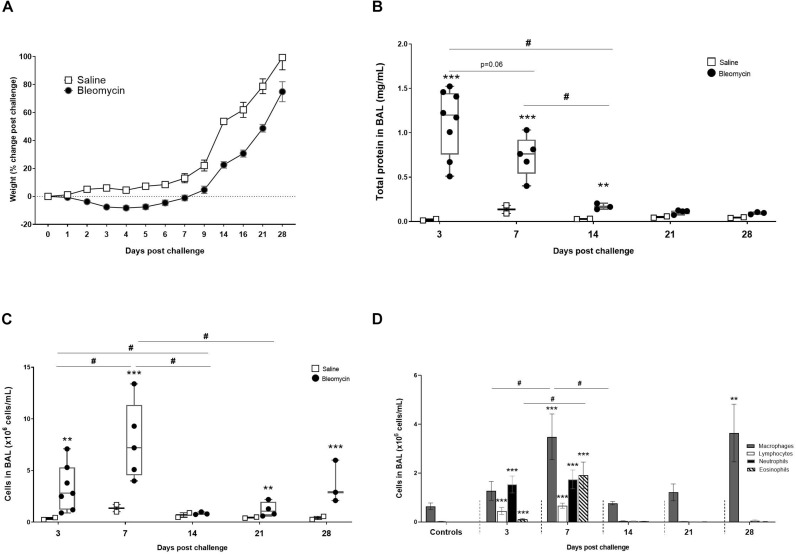
Characterization of the DIILD model, with bleomycin-induced lung injury. **(A)** Total body weight change in rats over time, expressed as% change compared to their baseline weight. From the BALF analysis total protein concentration and immune cell were assessed at different time points. **(B)** The total protein reflected plasma exudation and edema at initial inflammatory phase of the bleomycin model (day 3–7 post challenge). **(C)** The total cell count from BALF was peaking at day 7. Significant increase of cells was also observed at the latest time point (day 28), indicating a second phase of immune cell infiltration. **(D)** Differential cell counts indicating which cells dominate the various time points. The cells induced at the latest time point (day 28) are only macrophages although present at levels similar to the acute inflammation time point (day 7). Significance is indicated by ** when *p* < 0.01 and *** when *p* < 0.001. The comparison of various time points between bleomycin-challenged groups is expressed as # when *p* < 0.05.

Rats were monitored daily and body weight was documented. A decrease in bodyweight during the first days compared to baseline and saline controls was recorded (Figure 2A). Glucose levels are shown in Supplementary Figure S4. Blood glucose was generally slightly higher in the morning irrespective of the challenge group. However, during the inflammatory phase (days 3–7) animals challenged with bleomycin tended to have slightly lower blood glucose levels compared to the saline-challenged controls. No statistical differences were, however, recorded between groups or over time.

### Characterization of the Model

The lungs reacted rapidly to instillation of bleomycin. The total protein measurements from BALF propose increased plasma exudation already at day 3 post bleomycin challenge, when the maximal increase of total protein was observed (*p* < 0.001) compared to control (Figure 2B). Protein levels were lower in the bleomycin group at day 7 compared to day 3, but yet significantly higher (*p* < 0.001) compared to controls. Protein levels in lavage stayed significantly elevated for up to 14 days post challenge.

The cell analysis from BALF showed a significant increase in total cell count already after 3 days (*p* < 0.01), compared to saline control (Figure 2C). The cell count was further increased at day 7 compared to day 3 (*p* < 0.05), and also significantly higher (*p* < 0.001) compared to the saline control at this time point. After day 7, number of cells in lavage started to return to baseline, to increase again in a second peak of BAL cell counts on day 28.

The differential cell count indicates that different types of immune cells are activated and recruited to the lungs over time, starting with a predominantly neutrophilic influx at day 3, followed by an increase of macrophages, lymphocytes, and eosinophils on day 7 (Figure 2D). Macrophages are present throughout all time points, although both an increase in numbers as well as activation status can be seen at the peak of the inflammatory phase on day 7. A second macrophage induction was observed at later time points (day 28) being significantly elevated (*p* < 0.001) compared to control and previous measured levels at days 14 and 21 (Figures 2C,D).

### Histological Tissue Section Analysis

**FIGURE 3:**
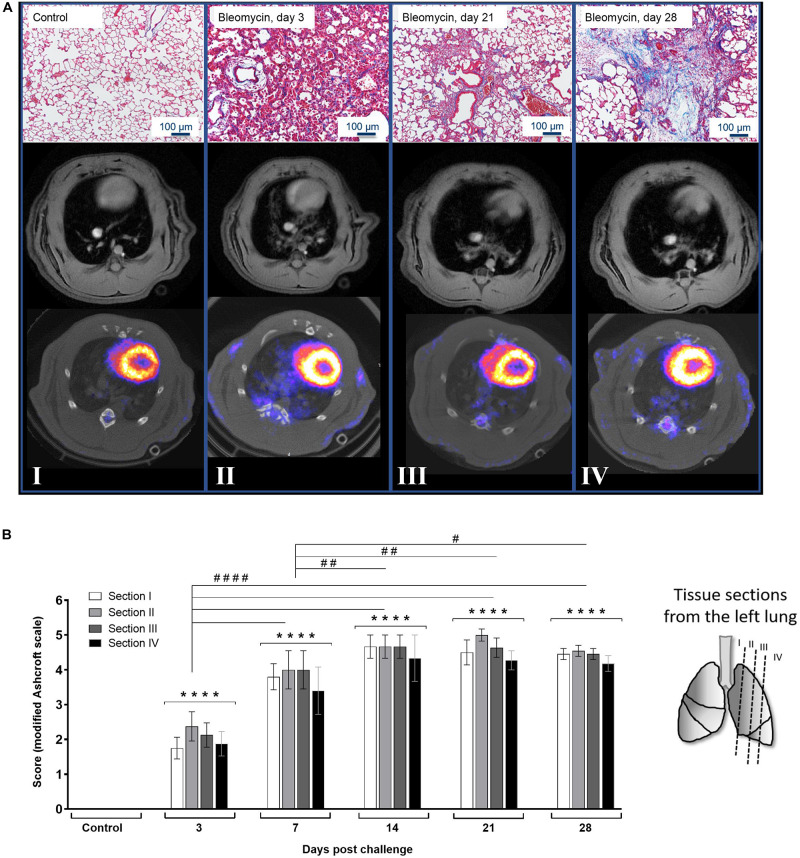
Histology and lung images at various time points post bleomycin challenge. **(A)** Representative images from Masson’s-Trichrome staining presented at baseline/control (I), day 3 (II), day 21 (III), and day 28 (IV) post bleomycin challenge, in association to MRI- and PET/CT images of the lungs. All images acquired during the imaging scan sessions were used for quantitative analysis, where data were extracted within the lung-ROI, which is defined in Supplementary Figure S2. **(B)** Scoring of the histological staining where histological sections were collected from different positions in the left lung lobe of the sacrificed rats. After applying the modified Ashcroft fibrosis score (scale ranging from 0 to 8), the scoring results indicate fibrotic progression over time while infiltration of inflammatory cells ceased and was cleared over time. Statistical comparison between sections at each time point was not significantly different, indicating even distribution of injury throughout all lung sections in each animal. Scoring variation between groups was compared and indicated by **** when *p* < 0.0001 when comparing bleomycin towards the saline control. The comparison of various time points between bleomycin-challenged groups is expressed as # when *p* < 0.05; ## when *p* < 0.01 and #### when *p* < 0.0001.

For every time point of imaging analysis, histology was assessed by staining lung tissue sections with Masson’s-Trichrome staining. As longitudinal imaging requires the same animals to be used throughout the study and histological analysis require termination, separate animals were used for the histological evaluation. These sections were qualitatively and quantitatively evaluated and presented as representative images in association with whole lung images obtained by MRI and PET/CT. Lesions can be observed by all techniques from the early inflammatory phase and onward (Figure 3A). The Masson’s-Trichrome stained tissue sections presented with increasing amount of blue stain with time, representing connective tissue accumulation (mainly collagen deposition), which becomes most prominent at the later time points. The sections were evaluated using the modified Ashcroft scale (Hubner et al., 2008). The score evaluation was performed for all samples, at all time points, and at four positions within each lung (Figure 3B). The modified Ashcroft score takes into account of cellular infiltrates and edema when evaluating alveolar wall thickness as well as fibrotic progression. The scoring reveals steadily increased injury over time, starting already at day 3 and continues, until reaching a plateau at day 28 with a score around 6 (on a scale of 0–8). The different sections (I–IV) at each time point, indicated a pattern pointing toward the lung injury being initiated centrally in the lung lobes (mainly sections II–III), and later spreads to the peripheral small airways (section IV) as well as to the large bronchi (section I), later on (Figure 3B). However, the statistical calculation did not reach significance when comparing sections within the same time points.

### Lung Volume and Lesion Volume Assessed by MRI

**FIGURE 4:**
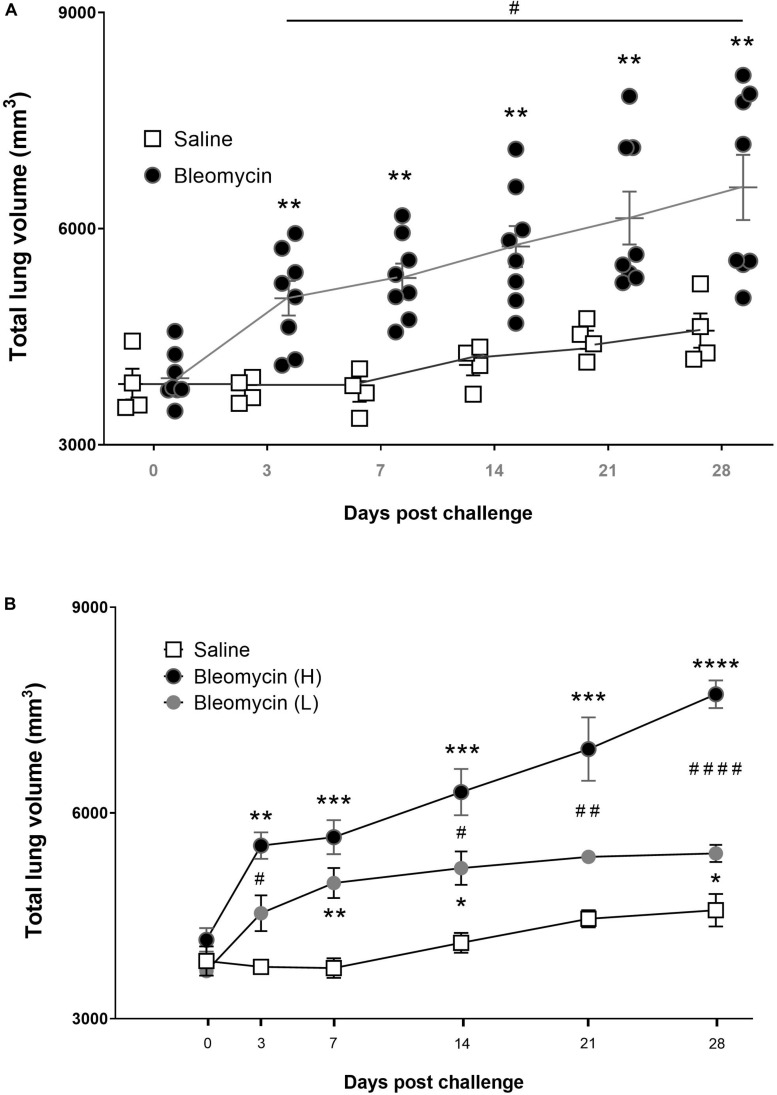
Lung volume changes post bleomycin challenge. **(A)** Total lung volume presented as mm^3^ in saline vs. bleomycin groups for all measured time points. The total lung volume data are generated from the MR-images with ROI, showing here that bleomycin challenge induced an increase in total lung volume compared to saline control. Later time points (day 21***–***28) revealed segmented populations of rats that responded differently, where the total lung volume change was further increased or recovered back toward baseline, being high- and low-responders, respectively. **(B)** Total lung volume change presented according to the different groups; saline, low-responders (L) and high-responders (H). Significance is indicated by * when *p* < 0.05; *p* < 0.01 by **; *p* < 0.001 by *** and *p* < 0.0001 by ****, when comparing bleomycin towards the saline control from the same time point. The comparison of various time points between bleomycin-challenged groups is expressed as # when *p* < 0.05; ## when *p* < 0.01 and #### when *p* < 0.0001.

Using MRI, it was noted that the lung volume increased slightly with time in all rats due to the growth of the animals. Additional increase of the total lung volume was observed in the bleomycin group, primarily during the inflammation phase during the first 3–7 days (Figure 4A). However, after the initial inflammatory phase (days 3–7), some animals returned to almost normal lung volumes indicative of resolution of disease. In other animals, lung volume did not return to baseline but instead continue to increase throughout the experiment, indicating progressive disease (Figure 4A). These two subgroups of animals were separated for further analysis based on this observation and split into groups “high-responders” (H) for animals with a progressive increase in total lung volume and “low-responders” (L) for those where total lung volume returned to normal at day 28 (Figure 4B).

**FIGURE 5:**
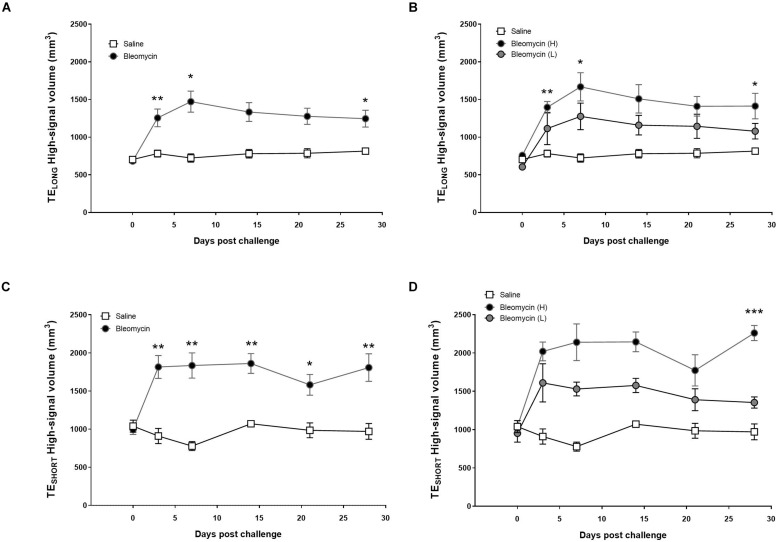
Observation of the high MRI signal from TE_LONG_ and TE_SHORT_ measurements, showing lesions from inflammation and fibrosis. **(A)** The high-MRI signal volume, applying TE_LONG_, in saline vs. bleomycin-challenged rats (**n** = 8). **(B)** When splitting the bleomycin group in high- vs. low-responders (**n** = 4 + 4), it is evident that the volume of assessed lesions is being reduced to similar levels as saline controls, while the high-responder group showed continuous large volume of the lesions at the last scan session (day 28). **(C)** Measuring the high signal throughout the various time points with the TE_SHORT_, displayed mainly fibrosis at later stage, in saline vs. bleomycin-challenged rats (**n** = 8). **(D)** The TE_SHORT_ measurement that identifies the high signal presented according to the split bleomycin groups in high- vs. low-responders (**n** = 4 + 4). Here the lesion volume is starting to recover toward saline controls in the low-responders, while the high-responder group showed progressive disease in forms of fibrotic lesions increasing significantly in volume, at the last scan session (day 28). Significance is indicated by * when *p* < 0.05; *p* < 0.01 by ** and *p* < 0.001 by ***, when comparing bleomycin towards the saline control from the same time point.

Subsequently, by using the histogram-based analysis of the total lung region (lung ROI), the “high-signal” from the MR-images could be presented, either on a group level including all bleomycin-challenged rats or with the high- and low-responder groups separately. The MRI measurements using TE_LONG_ showed that the high-signal volume in the lung is significantly higher in the bleomycin group than the control group at all time points post baseline, both when presenting the whole group (Figure 5A) and the separated bleomycin groups (Figure 5B). From the UTE of TE_SHORT_, the high-signal volume in the lung was significantly higher in the bleomycin group than the saline control group at all time points post baseline (Figure 5C). However, separating the high- and low-responders indicated striking difference at the last time point (Figure 5D). At day 28 post challenge, the high-signal lesions, measured by TE_SHORT_ sequence in the high-responder group revealed an increase of the lesion volume, significantly larger compared to control. Meanwhile the low-responder group returned back toward baseline at day 28.

Notably, the animals in the high-responder group tended to express a higher grade of disease for all relevant measurements, such as larger weight loss as well as differences observed in lung volume-to-bodyweight ratio, among other parameters, as can be observed in Supplementary Figures S5A,B. As the total lung volume was used as an IB indicating disease progression, as well as pointed toward two distinguished groups within the bleomycin-challenged group, additional short MRI-study was performed to confirm this phenomenon in a small group of rats, to see if the volume alteration assessed by MRI can be a reproducible IB. These supported data are presented in Supplementary Data File S2.

### PET Activity for Tracking Areas of High Glycolytic Metabolism

**FIGURE 6:**
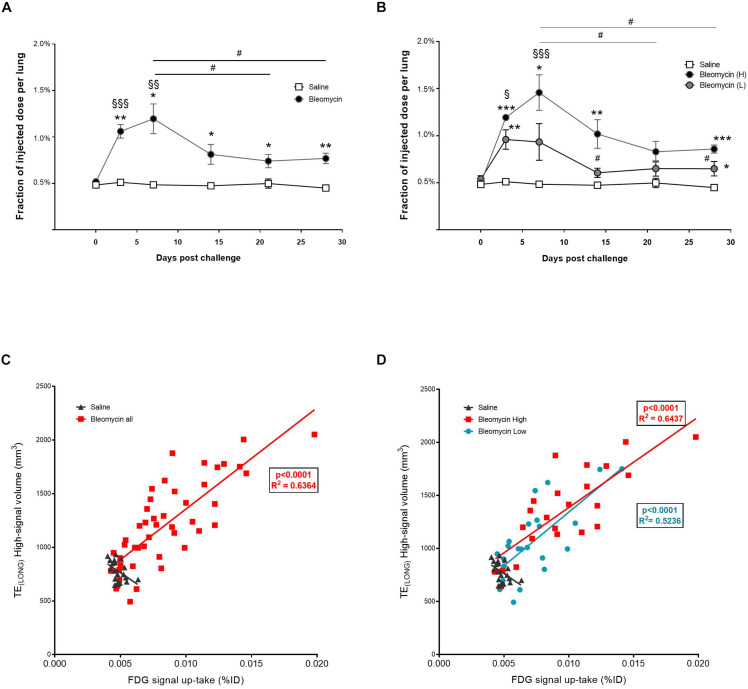
Quantification of PET-FDG signal up-take during inflammation and fibrosis. **(A)** Up-take of FDG-PET at different scan days, showed peak levels at inflammation (day 7), although does not return back to baseline values (saline) even though inflammation is not present during the fibrotic phase. Here the amount of animals was **n** = 4 in the saline group and **n** = 8 in the bleomycin group. **(B)** The signal peak of FDG-PET remains at day 7, also when presented according to the separated groups of high- and low-responders. Here the amount of animals was **n** = 4 in the saline group and **n** = 4 in each bleomycin group. Significance is indicated by * when *p* < 0.05; *p* < 0.01 by ** and *p* < 0.001 by ***, when comparing bleomycin towards the saline control from the same time point. The comparison of various time points between bleomycin-challenged groups is expressed as # when *p* < 0.05, while bleomycin group compared towards baseline (within the same group of animals), significance is indicated by § when *p* < 0.01; *p* < 0.01 by §§ and *p* < 0.001 by §§§. **(C)** PET signal up-take correlate with MRI signal (TE_LONG_), presented within the whole bleomycin-challenged group. The red line in the graph is a linear fit to data from all animals, the **R**^2^ value is for this fit (**R**^2^ = 0.6364). **(D)** When presenting the separate groups of high- and low-responders, still significant correlation is observed. The red line in the graph is a linear fit to data from the high-responder group (**R**^2^ = 0.6437) while the blue line is linear fit to data from the low-responder group (**R**^2^ = 0.5236). For the statistical analysis, Spearman’s rank correlation was applied.

FDG-PET signal up-take was observed mainly during the inflammatory phase of bleomycin-induced lung injury, with a peak at day 7, although stayed significantly elevated also at later time points, compared to control (Figure 6A). The total up-take of FDG-PET in the lung was significantly higher in the high-responder group compared to the control group at all time points, except at 21 days post instillation. The mean up-take of FDG-PET was significantly higher in the high-responder bleomycin group compared to baseline values. The increased FDG-PET signal up-take in the high-responder group stayed elevated for longer (day 14) and in addition seemed to increase again at day 28 (Figure 6B). This is the similar pattern as observed for total lung volume changes and lesion volume measurements by MRI, and in particular by TE_LONG_. The total up-take of FDG in the lung correlates significantly (*p* < 0.001) with high MRI signal volume from TE_LONG_ observed from all rats that received bleomycin (Figure 6C). Correlation was also observed when comparing the groups based on the separate high- vs. low-responder groups (Figure 6D). In Supplementary Figure S6A, the two different scans using TE_LONG_ and TE_SHORT_ are presented in a bleomycin-challenged rat compared to a control rat. Also, in Supplementary Figure S6B, merged images can be observed from the different imaging modalities.

### Regulation of Inflammatory and Pro-fibrotic Genes Over Time in Bleomycin-Challenged Rats

**FIGURE 7:**
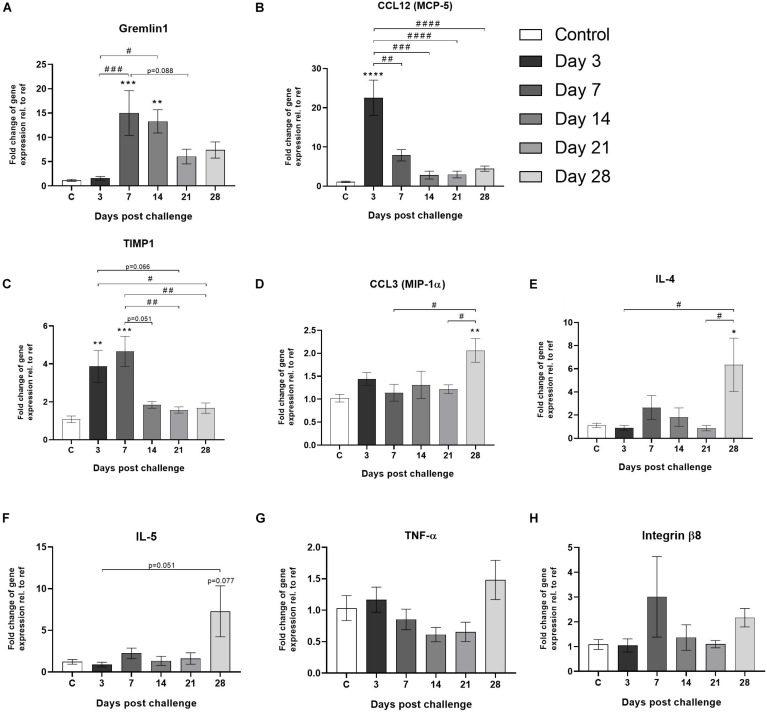
Gene expression of markers involved in lung injury and wound healing. Gene expression of **(A)** gremlin1, **(B)** CCL12 (MCP-5), **(C)** TIMP1, **(D)** CCL3 (MIP-1α), **(E)** IL-4, **(F)** IL-5, **(G)** TNF-α, and **(H)** integrin-β8, are all presented as mean ± standard error of the mean (SEM). All samples from the bleomycin group are related to the mean of control and normalized to the geometric mean of five different reference genes. Each presented group/time point contains **n** = 4***–***8. Significance is indicated by * when *p* < 0.05; *p* < 0.01 by **; *p* < 0.001 by *** and *p* < 0.0001 by ****, when comparing bleomycin towards the saline control. The comparison of various time points between bleomycin-challenged groups is expressed as # when *p* < 0.05; ## when *p* < 0.01; ### when *p* < 0.001 and #### when *p* < 0.0001.

In order to generate a comprehensive gene expression analysis over time, animals not included in the longitudinal imaging groups were terminated at day 3, 7, 14, 21, and 28 and lungs harvested for molecular analyses. Gene expression analysis from frozen lung tissue revealed interesting kinetic patterns over time. Early on (days 3–7), expression of inflammatory related gens such as gremlin1, CCL12, and TIMP1 were observed to be significantly upregulated (Figures 7A–C), whereas at later time points CCL3, IL-4, and IL-5 (Figures 7D–F) were increased. TNF-α seemed to increase slightly at day 28 (Figure 7G) while integrin-8β presented with an early (day 7) and one late (day 28) upregulation compared to control (Figure 7H).

**FIGURE 8:**
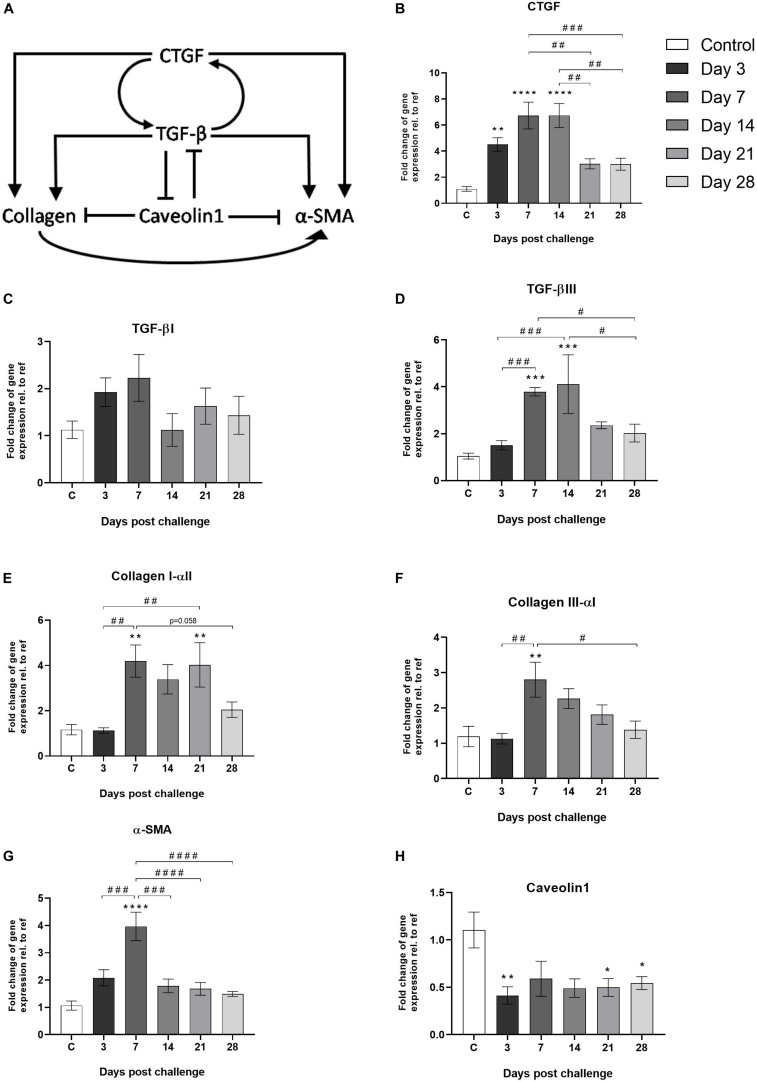
Gene expression of markers known to be involved in the TFG-β/CTGF-axis. **(A)** Schematic image of the TGF-β/CTGF-axis involving markers known to be part of the fibrosis process in lung tissue (Wang et al., 2006; Lipson et al., 2012; Lin et al., 2013). Gene expression of **(B)** CTGF, **(C)** TGF-βI, **(D)** TGF-βIII, **(E)** Collagen I-αll and **(F)** Collagen llI-αl, **(G)** α-SMA, and **(H)** Caveolin1, are presented as mean ± standard error of the mean (SEM). All samples from the bleomycin group are related to the mean value of all control samples and normalized to the geometric mean of five reference genes. Each presented group/time point contains **n** = 4***–***8. Significance is indicated by ** when *p* < 0.01; *p* < 0.001 by *** and *p* < 0.0001 by ****, when comparing bleomycin towards the saline control. The comparison of various time points between bleomycin-challenged groups is expressed as # when *p* < 0.05; ## when *p* < 0.01; ### when *p* < 0.001 and #### when *p* < 0.0001.

For pro-fibrotic markers, genes linked to the TGF-β/CTGF-axis were analyzed (Figure 8A). The up-stream cytokine CTGF was shown to increase at early time points (day 3) and stayed significantly elevated until day 14 (Figure 8B). In addition, TGF-β (Figures 8C,D), Collagen type I and III (Figures 8E,F), as well as smooth muscle actin (α-SMA) were significantly increased already during the inflammatory phase in the bleomycin-challenged group, compared to control (Figure 8G). At the same time, the negative regulator of TGF-β; caveolin1, was downregulated upon bleomycin-challenge (Figure 8H).

### Genes Altered at Day 28—Comparison of High- vs. Low-Responder Groups

**FIGURE 9:**
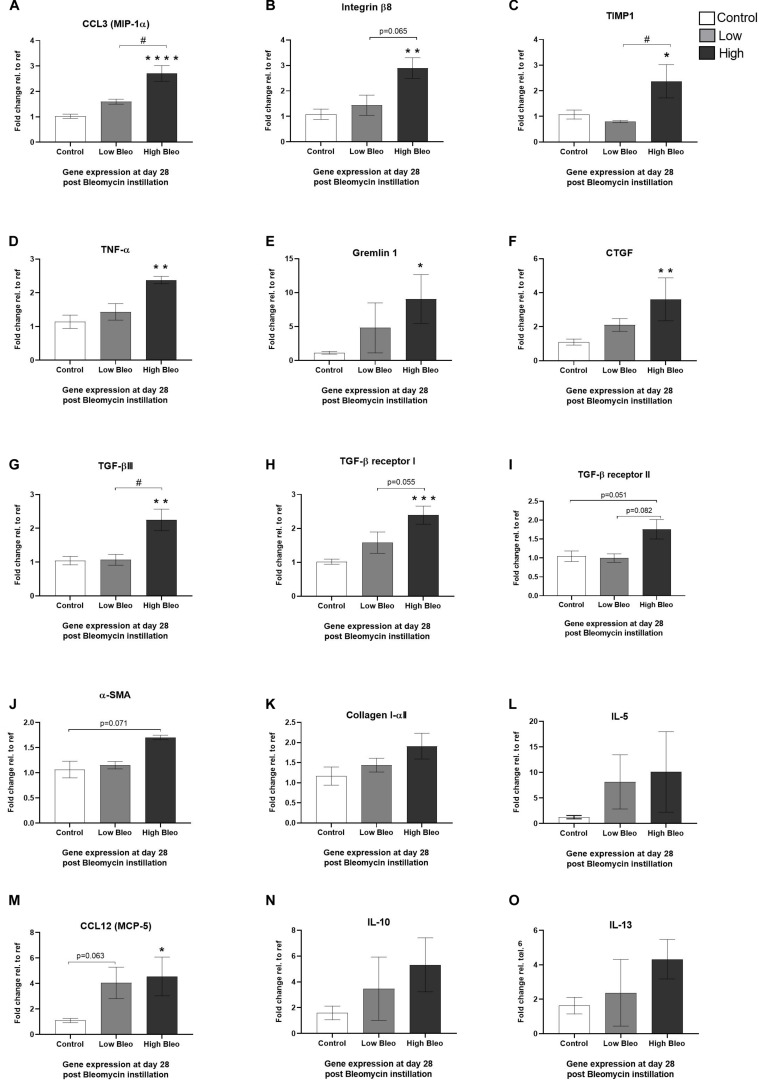
Gene expression at day 28 in samples from high- and low-responder groups. Gene expression of **(A)** CCL3 (MIP-1α), **(B)** integrin-β8, **(C)** TIMP1, **(D)** TNF-α, **(E)** gremlin1, **(F)** CTGF, **(G)** TGF-βIII, **(H)** TGF-β receptor I, **(I)** TGF-β receptor II, **(J)** α-SMA, **(K)** Collagen I, **(L)** IL-5, **(M)** CCL12 (MCP-5), **(N)** IL-10, and **(O)** IL-13, are all presented as mean ± standard error of the mean (SEM). All samples from the bleomycin group are related to the mean of control and normalized to the geometric mean of five different reference genes. Each presented group contains **n** = 3 and are only samples analyzed from the group of rats undergoing longitudinal imaging, being terminated at day 28. Significance is indicated by * when *p* < 0.05; *p* < 0.01 by ** and *p* < 0.001 by ***, when comparing bleomycin towards the saline control. The comparison between bleomycin-challenged groups is expressed as # when *p* < 0.05 and #### when *p* < 0.0001.

At termination of the longitudinal imaging, lungs were harvested and gene expression was analyzed from lung tissue samples collected at day 28. In this analysis, it was also possible to compare expression profiles between high- and low-responders with respect to MRI total lung volume as described above. Several interesting genes were significantly increased in the high-responder group compared to control, whereas the low-responder group showed little or no upregulation. Significant differences were seen for some of the more inflammatory related genes as identified above, i.e., CCL3, integrin-β8, TIMP1, TNF-α, and gremlin1 (Figures 9A–E) but also the pro-fibrotic genes such as CTGF, TGF-βIII, and the TGF-β receptor (Figures 9F–I). On the other hand, genes that did not increase differently among the bleomycin groups (high vs. low) compared to control were α-SMA, collagen I, IL-5, CCL12, IL-10, and IL-13 (Figures 9J–O).

### Investigated Immune Cells and Markers During Lung Injury

**FIGURE 10:**
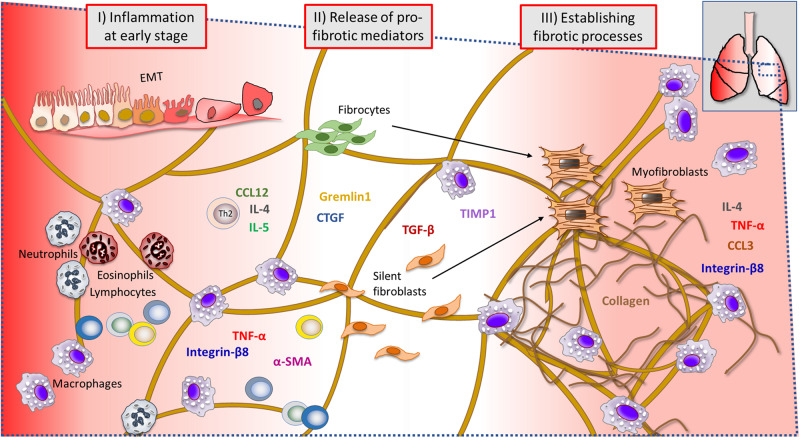
Summary of events during ongoing lung injury, with related cells and markers involved at the injury site of the lung tissue. Immune cells and gene markers that were analyzed throughout this study are presented in a probable scenario during lung injury, at a microscopic viewed site within the lung tissue. The early phase of bleomycin-triggered injury induced immune cell activation and infiltration, observed significantly elevated at day 3–7 post challenge (I). Simultaneously, inflammatory cytokines and pro-fibrotic markers assessed to be significantly increased at this point were several important markers known to be involved in driving the fibrosis progression (II). The assessed lesions by histology, indicated collagen-rich sites and dense fibrotic lesions, also evident by MRI and PET imaging. The later time points (III) indicated a second phase of macrophage infiltration (day 28) in BAL, while several genes were upregulated in particular during the last day of this model (EMT, epithelial–mesenchymal transition).

During lung injury, many events are ongoing at the same time (Figure 10). As indicated by our results, the early phase post bleomycin challenge involved immune cell activation and infiltration being prominent at day 3–7 post challenge (I). At the same time, many inflammatory cytokines and pro-fibrotic markers are being released which was observed by significant increase of total proteins in BALF and also gene expression upregulation of many important markers driving the fibrosis progression (II). At later time points (III) fibrotic foci were assessed by MRI and PET imaging but also by histology, which indicated collagen-rich sites and dense fibrotic lesions. BAL cell count indicated a second phase of macrophage infiltration (day 28), while several genes were upregulated in particular during the last day of this model.

## Discussion

Using the bleomycin rat model, we have studied MRI and PET IBs for inflammation and fibrosis. In addition, non-IBs were used to further characterize the model and to support the imaging results. The findings were validated with histology. As previously reported, we noted an initial weight loss in the bleomycin-challenged group, cellular inflammation, and plasma exudation as measured by cells and protein content in BALF at the early phase, i.e., during the first week post challenge. This is in agreement with previous studies (Izbicki et al., 2002; Walters and Kleeberger, 2008; Bethany et al., 2013; Reinert et al., 2013; Della Latta et al., 2015). Fibrotic changes are normally expected to start around day 14 and peak around day 21 (Izbicki et al., 2002; Bethany et al., 2013; Reinert et al., 2013). However, in our study, the fibrosis was still progressing at least up to day 28 as indicated by the modified Ashcroft score and imaging data. Based on our findings, we can conclude that appropriate time points were selected for termination and imaging sessions in order to explore various imaging parameters with the aim to study inflammatory and fibrotic lesions.

An increasing number of clinical studies are considering MRI as the future preferred modality for longitudinal lung injury assessment, although there is a need for further optimization to enable robust protocols and user settings (Barreto et al., 2013). In this study, we explored MRI and FDG-PET for longitudinal monitoring of lung lesions after bleomycin challenge. Initially, we observed that total lung volume, measured by MRI, increased significantly over time in the group of rats receiving the bleomycin challenge. An increase of total lung volume has been previously observed, and is used as an IB in the clinic for identifying lung injury and progressive lung diseases characterized by hyperinflation (Gibson, 1996; Ferguson, 2006). Previously, increased total lung volume has also been observed in preclinical studies where emphysema and as well as fibrosis was studied in rats (Egger et al., 2014; Vande Velde et al., 2014, 2015; Bianchi et al., 2015). The increase in total lung volume in animal disease models as well as in clinical observations is probably due to compensatory mechanisms dependent on the mechanical regulation of respiratory reflexes (Hirai et al., 1999; Leith and Brown, 1999; Ferguson, 2006). However, the increase in volume does not necessarily reflect the quality or the functional volume of the lung but may simply be explained by static (loss of recoil) and dynamic (air trapping) effects resulting in hyperinflation, as is most often evident in clinical COPD (Ferguson, 2006). Although the increase of total lung volume in our experiments was only modest, it was significantly increased compared to the control group. All rats that received bleomycin presented with increased lung volume, and the largest increase occurred during the first week of the model, indicating compensatory mechanisms due to the acute bleomycin induced injury. Even though clinical ILD is a restrictive lung disease and manifests mainly by reduced lung volume capacity, the lung volume increase in animal models such as in the bleomycin model has been used in numerous models over the past years (Egger et al., 2014; Vande Velde et al., 2014, 2015; Bianchi et al., 2015). Therefore, lung volume was employed as a well-known biomarker to further assess other parameters from our imaging data. From these changes in total lung volume, we were able to separate the animals into two distinct groups possibly representing different disease severity, and hereafter referred to as high- and low-responders. The high responders had a continuously high total lung volume all the way up to day 28, when the initial inflammation had resolved. The high responder group could represent animals progressing into more severe disease and possibly chronic fibrosis. The two groups identified by use of total lung volume also demonstrated different levels of lesions as identified by MRI and PET.

In an attempt to differentiate between inflammation and fibrosis, MRI sequences of TE_LONG_ (inflammation) and TE_SHORT_ (fibrosis + inflammation) UTE were evaluated, but they did not provide as distinct differentiation between the two disease states as expected. More work will be required to optimize the current protocol in order to generate discrete patterns of inflammation and fibrosis using this imaging sequence with two TEs. A complication of the bleomycin model, which ideally results in initial inflammation followed by fibrosis, is that these two disease characteristics are more or less simultaneously present during the time frame of the disease progression studied (Moeller et al., 2008). Figures 5A,C reflect the concern of overlapping pathologies simultaneously. This is indicated by the peak of TE_LONG_ at day 7 also overlapping in time when lesion signal is evident by TE_SHORT_ on days 3–14. The fibrotic changes may therefore be underestimated at the later time points also caused by differences within the group of animals. However, the results presented for high- vs. low-responder groups may represent the two different outcomes of progressive disease and resolution, as evident in Figures 5B,D. We believe that lesions with increased signal, when imaged using TE_SHORT_ at day 28, may indicate an increasing fraction of remodeling being present, while inflammation is reduced and slowly resolving as illustrated by TE_LONG_ signal going down. This implies that a more severe progressive remodeling may be induced in the high responder group, whereas the others may start to resolve the disease. This is also supported by the histology and BAL data, showing reduced inflammation while increased degree of fibrosis over time, post bleomycin instillation. Even if the two different TE sequences may not be fully optimized in order to differentiate inflammation from fibrosis, they could still contribute to increase our understanding about both the model as well as the applied imaging modalities.

FDG-PET is an established IB in the clinic mainly to identify tumors or chronic inflammation (Glaudemans et al., 2013) and is also regularly used for measurements and localization of inflammation in preclinical models (Bondue et al., 2015,2019). In our study, the FDG-PET signal up-take peaked as expected at day 7, during the inflammatory phase. There was a positive correlation between total FDG up-take and MRI with TE_LONG_. The inflammation was also confirmed by histology as well as cells and protein in the lavage. However, the signal up-take of FDG-PET continued to be elevated compared to baseline, even when inflammation decreased. This has been observed also in a previous study (Bondue et al., 2015), indicating that FDG-PET could be used as a biomarker of fibrosis as well as inflammation, even though fibrosis has not been described to show as high energy metabolism as inflammation. In fact, recent clinical studies indicate similar findings, and demonstrate the potential utility of FDG-PET in predicting clinical prognosis in IPF patients (Groves et al., 2009; Justet et al., 2017). With respect to clinical use in early detection of DIILD, FDG-PET is already used in routine follow-up of patients with Hodgkin’s disease where it is used to monitor disease progression. As these patients are often treated with drugs with a high incidence of causing DIILD, such as bleomycin, the possibility of detecting both the inflammatory and fibrotic stages of DIILD makes FDG-PET an attractive IB for use in these patients (Paschali et al., 2017). Even though FDG-PET may not be specific IB of fibrosis and the fibrotic phase is not described to show as high energy metabolism as during inflammation in the lung, the glycolytic metabolism could still be targeted in the future by either a non-specific tracer such as FDG-PET or aim at developing a rather specific target within the glycolysis cycle. Irrespective, it has been suggested that this could potentially be the Warburg effect (Chen et al., 2018) occurring at the site of active fibrosis formation. The potential of targeting this mechanism could be further developed (Maher, 2015). If fibrosis only, rather than a mixture of inflammation and fibrosis is the subject of interest in a DIILD related examination, one could instead aim for a more specific marker targeting fibrosis. Recently, fibrosis targeting probes for PET imaging have been developed, such as small peptides targeting newly synthesized collagen (Desogere et al., 2017a, b) or using the MRI probe Gd-Hyd that is able to specifically detect lysyl oxidase that is essential for crosslinking of collagen (Chen et al., 2017).

Combining MRI and FDG-PET could offer an improved way to follow development and progression of DIILD. If robust IB could be established from well-characterized models as our study shows with confirmed pathology by cells and histology, the IB could then be used to develop new additional animal models or investigating new drugs. As a result, both new DIILD models as well as novel IB could be developed. However, irrespective of which drug class that is investigated and what ILD pathology that can be mimicked *in vivo*, there is still a need for better imaging protocols and data analysis tools. Our method to define “high MRI signal” volume as the lesion, is histogram-based and avoid subjective decisions similar to the Lloyd-Max histogram quantitation used by many studies previously (Babin et al., 2011; Egger et al., 2013, 2014, 2015, 2017). We did not utilize the specific intensity of the MRI signal beyond finding a threshold, partly because we have not utilized an in-view standard to normalize the signal across measurement, as has been done in some other studies that include lung imaging (Shioya et al., 1993; Tournebize et al., 2006; Strobel et al., 2012).

The strength of this study is that a multi-imaging modality approach was applied, and the MRI and PET were performed in the exact same geometry during continuous anesthesia. In this way, the images from the two modalities of the same animal and day could be analyzed without registration. A limitation of this study is the model design, where one dose of bleomycin in a single administration is given to the animals intratracheally. This is a well-documented model; however, it does not perfectly reflect the human conditions in DIILD. Drug administration to the patient in the clinical setting occurs systemically and the drug is often taken at several occasions. This enables withdrawal of the drug, if adverse effects are suspected or detected. Therefore, a chronic exposure model may be of interest for future DIILD studies (Headley et al., 2018). In addition, a chronic model may facilitate the development of improved IB that better can distinguish pro-fibrotic processes, from lesions containing inflammatory infiltrates and edema due to the vascular leak.

Very few preclinical imaging studies of lung injury have been reported, in which the explicit aim has been to study DIILD with a dedicated scope to evaluate adverse effects from drug administration. Even fewer studies have focused on the translational aspects with transferable imaging protocols and experimental settings that can be applied within the clinic (Skeoch et al., 2018). The longitudinal approach of this study is clinically relevant, as longitudinal follow-up imaging over time and the use of non-invasive imaging methodology could improve clinical management of DIILD. We can see that there may be a need for even longer follow-up, and to follow the same animals by, e.g., imaging and regular blood sampling. With our longitudinal and non-invasive imaging approach, we were able to find that all rats presented with inflammation in the lung at the early stage, while only half of the group continued to progress toward fibrosis. These results are in line with how clinical DIILD can manifest itself differently in patients.

In addition to the longitudinal imaging follow-up, we were also able to map the gene expression profile for a number of key markers over time. Important cytokines in wound healing such as CTGF and TGF-β are initiated early on, already during the peak of inflammation, as are key markers of fibrotic changes, such as actin and collagen. Other early induced markers observed in this study were CCL12 and gremlin1, which have been described as pro-fibrotic markers. CCL12 plays an important role in the recruitment of fibroblast in lung fibrosis (Bethany et al., 2013) and gremlin is found to be overexpressed in fibrotic disease (Koli et al., 2006). The gene profile points toward a dramatic change in the inflammatory process during the late phase of this model. Interestingly, markers such as IL-4 and CCL3 were significantly increased at day 28 compared to control. IL-4 has previously been shown to play an important role in lung injury and fibrosis when initiated at late stage (Huaux et al., 2003). As previously published data suggest crucial role for CCL3 in cell recruitment of macrophages and fibrocytes in bleomycin-induced fibrosis (Ishida et al., 2007). In our study, CCL3 might be one of several cytokines acting as chemotactic protein for cell recruitment in the late stage (day 28), as evident by the second phase of BAL cell recruitment presented in Figures 2C,D. This second peak of inflammatory cells in BAL at day 28 coincides with an increased MRI signal at TE_SHORT_ and PET-FDG signal up-take in the lungs at later time points. In conclusion, several important inflammatory and pro-fibrotic markers were assessed that have increased our understanding of the kinetics of disease progression over 28 days. However further gene markers may be investigated to map the complete immune cell movement and activity in inflammatory or fibrotic sites.

## Conclusion

In summary, we were able to show significant differences between the inflammatory and the fibrotic phase of bleomycin-induced lung injury using traditional invasive read-out such as histology, BAL, and gene analyses. Using MRI and FDG-PET imaging, we were also able to demonstrate significant lung alterations in the bleomycin-challenged animals. In addition, two sub-groups representing high- and low-responders could be distinguished using MRI. The two MRI UTE sequences, TE_SHORT_ and TE_LONG_, could not fully differentiate between different pathologies and further optimization would be required. However, the group identified as high responders had a significantly higher lesion volume using TE_SHORT_ at day 28 post challenge as compared to control, whereas the low-responders at this time point had returned to baseline and were not different to the control group. Future assessment of lesions in preclinical DIILD or other lung injury models would benefit from robust and translational IB, providing potential for clinical use. Future implications would then improve the outcome for patients if new IB could detect and distinguish fibrosis from inflammation. Knowing the disease state of the patient could enable selection of more optimal treatment regimes. An additional application for future IB in lung injury models would entail opportunities for the pharmaceutical industry to implement these IB into preclinical drug safety assessment.

## Data Availability Statement

The datasets generated for this study are available on request to the corresponding author.

## Ethics Statement

The animal study was reviewed and approved by Ethical Committee Lund/Malmö, Sweden, with permit numbers 4003/2017 and 3226/2017.

## Author Contributions

IM, KW, and LO conceived the design of this study. KW and LO supervised the scientific work. IM, HF, and JL performed the experiments. IM, HF, AÖ, and KW analyzed the data. IM, HF, AÖ, KW, and LO contributed to interpretation of the data. IM drafted the manuscript. IM, HF, and AÖ produced the figures and tables. All authors were responsible for intellectual input and approved the final version.

## Conflict of Interest

HF, JL, and KW were employed by the company Truly Labs. The remaining authors declare that the research was conducted in the absence of any commercial or financial relationships that could be construed as a potential conflict of interest.

## Footnotes

1 www.invicro.com

2 www.graphpad.com

## Supplementary Material

FIGURE S1 Imaging modalities combined during scan sessions, with MRI-PET and CT. **(A)** Images of the MRI system and **(B)** 3D-printed animal bed connections enabling imaging with both MRI and PET/CT systems. **(C)** PET/CT system and **(D)** in close-up view, the animal bed used for both MRI and PET/CT scans.

FIGURE S2 Regions of interest (ROI). All slices (0–16 sections) of one rat lung in the transverse plane starting from the superior sections, generated from one MRI scan. Drawn ROI area indicated in red.

FIGURE S3 Histogram analysis within the region of interest (ROI). **(A)** Schematic image explaining how the histogram analysis method was done. Intersection of the slope at the x-axis is where the boarder is set, serving as the threshold value between the normal signal vs. high-signal within the lung. Controls (Saline) and bleomycin challenged lungs exert somewhat different shape of the histograms generated from the lung ROI. **(B)** Images of representative MR-scans from two rats, one from each group of saline (I) and bleomycin (II). After drawn ROI of the lungs (red area) in the same rat from saline (III) and bleomycin (IV) with cyan colored areas indicating “high MR-signal”, segmented by using the histogram thresholding analysis. High-signal signified lesions and vessels within the lungs (or only vessels in control animals).

FIGURE S4 Glucose levels monitored before each imaging scan. Blood glucose measurements before each scan session from day 3 **(A)**, 7 **(B)**, 14 **(C)**, 21 **(D)** and 28 **(E)**.

FIGURE S5 Body weight and disproportional lung volume increase in bleomycin-challenged rats. Body weight **(A)** and lung-ratio presented **(B)** according to the split bleomycin-challenged group into low- vs. high-responders to bleomycin-induced lung injury. Lung ratio was generated by calculating lung volume per body weight at that particular scan session, and presented as mean ratio for the whole group Significance was indicated by * when *p* < 0.05; *p* < 0.01 by ** and *p* < 0.001 by *** when comparing bleomycin towards the saline control from the same time point. The comparison of various time points between bleomycin-challenged groups is expressed as # when *p* < 0.05 and ## when *p* < 0.01.

FIGURE S6 Representative images of MRI echo times and multimodality images overlapping. **(A)** two different scans using TE_LONG_ and TE_SHORT_ are presented in a bleomycin-challenged rat and one saline control rat. **(B)** Overlap images from both PET and MR, demonstrating how the different layers of images and ROI could be aligned. This method enables data extraction within the same ROI generated. At the same time images acquired by different techniques can be complementary when assessing lesions.

DATA FILE S1 Extended section of the Materials and Methods. Study severity and adverse effects were described according to the ARRIVE guidelines, as well as the power calculations and n-numbers.

DATA FILE S2 Supporting material of the total lung volume from a pilot study. The total lung volume increase was observed in another experiment and set of animals, indicating the robustness of this imaging biomarker.

## Abbreviations

BALF: bronchoalveolar lavage fluid
CCL: chemokine (C-C motif) ligand
COPD: chronic obstructive pulmonary disease
CT: computed tomography
CTGF: connective tissue growth factor
DIILD: drug-induced interstitial lung disease
ECM: extracellular matrix
FDG: fludeoxyglucose
FOV: field of view
HRCT: high resolution computed tomography
IB: imaging biomarker
IL: interleukin
ILD: interstitial lung disease
IPF: idiopathic pulmonary fibrosis
i.t.: intratracheal
LBIC: Lund University BioImaging Centre
MLEM: maximum likelihood estimation method
MRI: magnetic resonance imaging
PET: positron emission tomography
ROI: region of interest
TGF- β: tissue growth factor β
TIMP1: tissue inhibitor of metalloproteinase 1
TNF- α: tumor necrosis factor α
TR: repetition time
TRISTAN-IMI: Translational Imaging in Drug Safety Assessment-Innovative Medicines Initiative
UTE: ultra short echo time.
