# Supplementary figures and images for: Imaging Biomarkers and Pathobiological Profiling in a Rat Model of Drug-Induced Interstitial Lung Disease Induced by Bleomycin
Source: Front Physiol. 2020 Jun 19;11:584. doi: 10.3389/fphys.2020.00584 (PMC7317035; doi:10.3389/fphys.2020.00584)

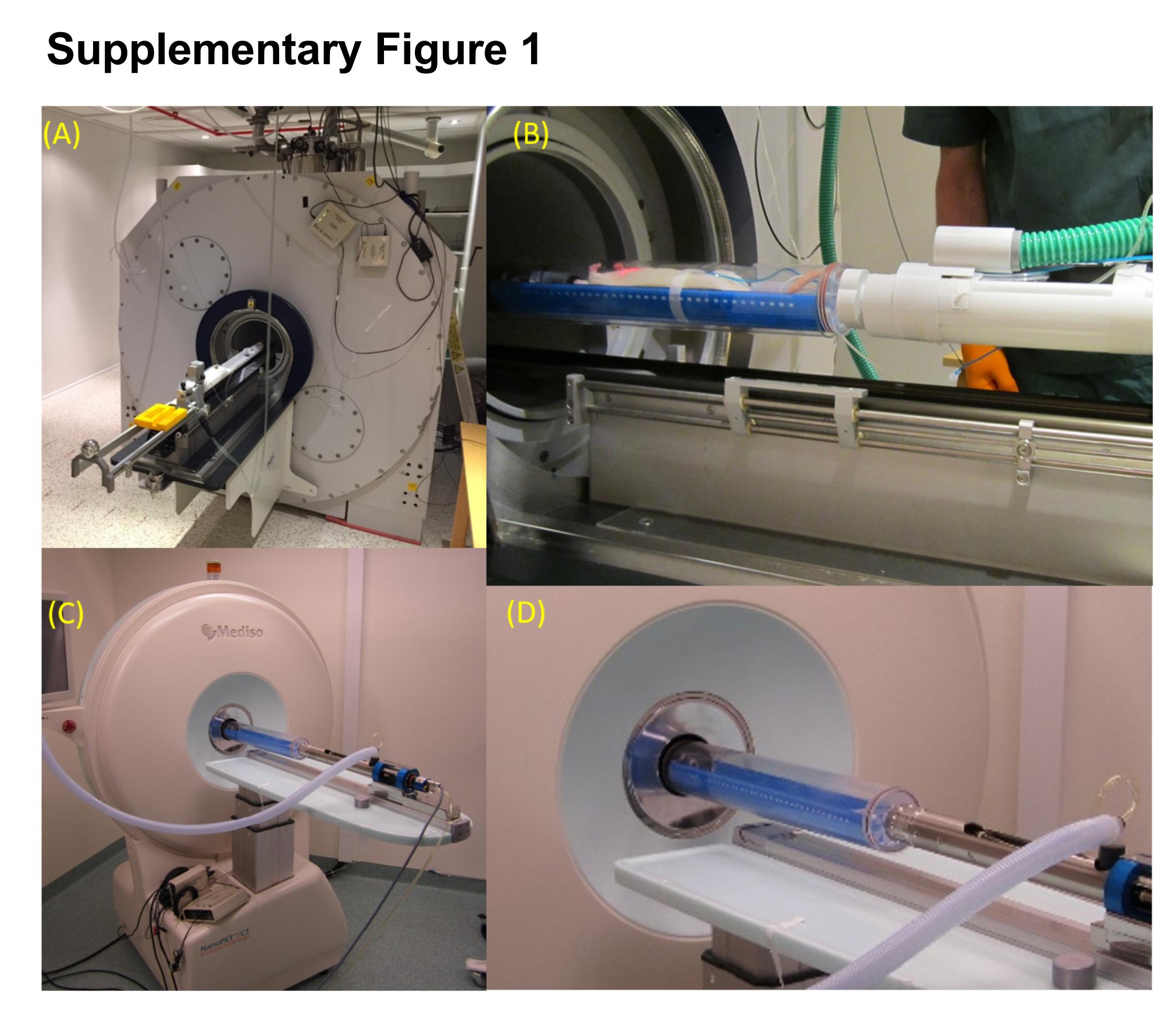

Supplement: FIGURE S1 — Imaging modalities combined during scan sessions, with MRI-PET and CT. (A) Images of the MRI system and (B) 3D-printed animal bed connections enabling imaging with both MRI and PET/CT systems. (C) PET/CT system and (D) in close-up view, the animal bed used for both MRI and PET/CT scans. [file Image_1.JPEG]

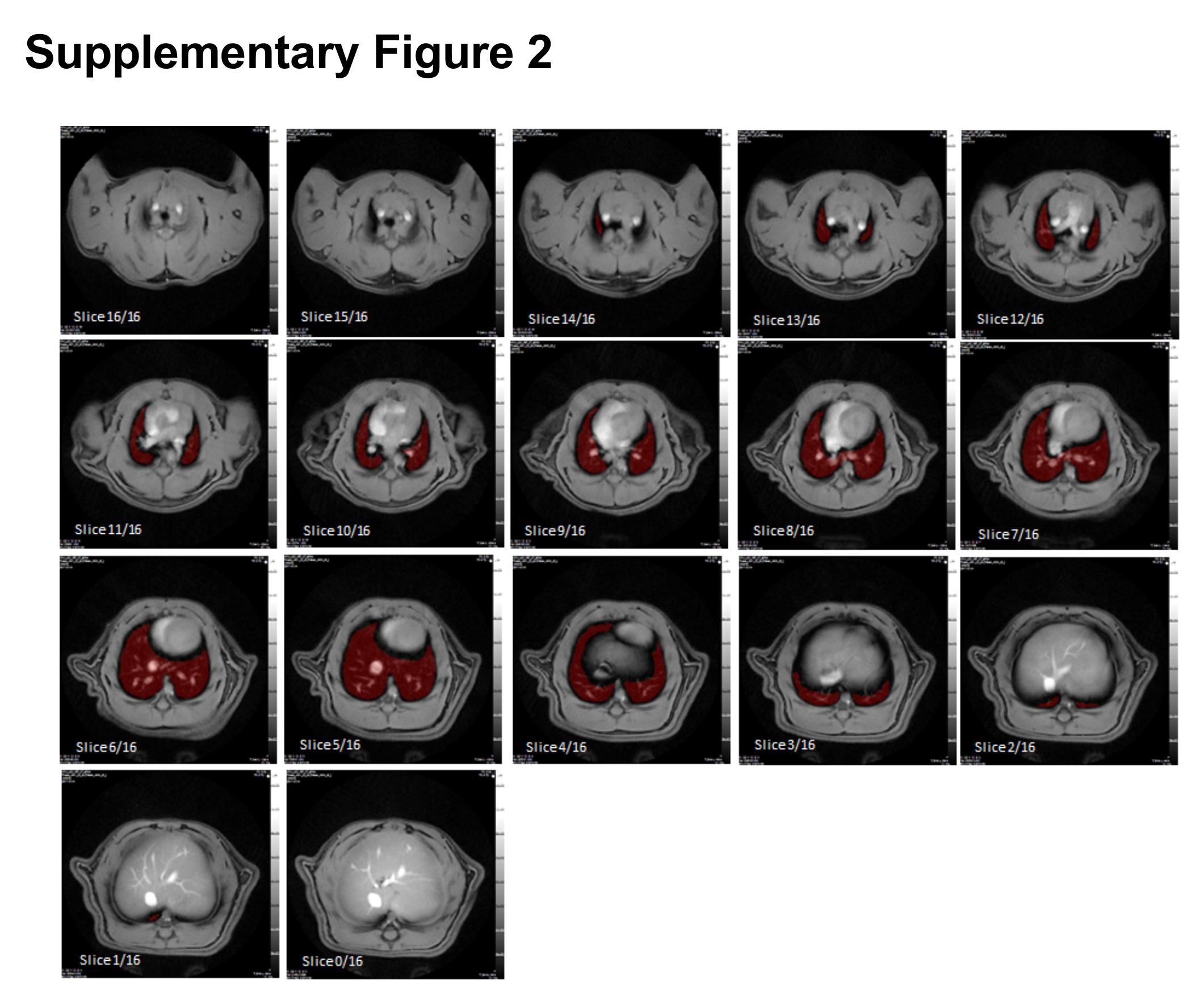

Supplement: FIGURE S2 — Regions of interest (ROI). All slices (0–16 sections) of one rat lung in the transverse plane starting from the superior sections, generated from one MRI scan. Drawn ROI area indicated in red. [file Image_2.JPEG]

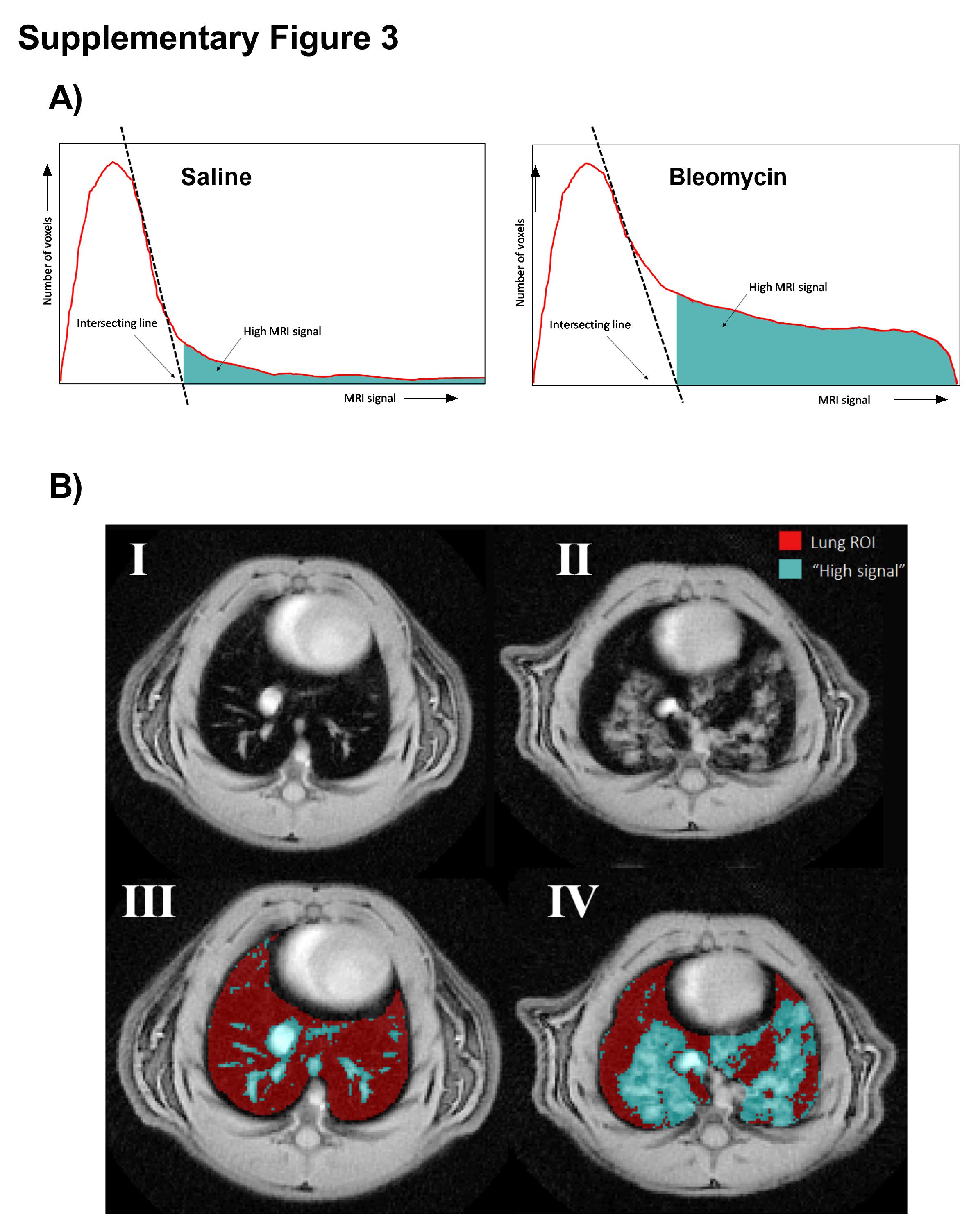

Supplement: FIGURE S3 — Histogram analysis within the region of interest (ROI). (A) Schematic image explaining how the histogram analysis method was done. Intersection of the slope at the x-axis is where the boarder is set, serving as the threshold value between the normal signal vs. high-signal within the lung. Controls (Saline) and bleomycin challenged lungs exert somewhat different shape of the histograms generated from the lung ROI. (B) Images of representative MR-scans from two rats, one from each group of saline (I) and bleomycin (II). After drawn ROI of the lungs (red area) in the same rat from saline (III) and bleomycin (IV) with cyan colored areas indicating “high MR-signal”, segmented by using the histogram thresholding analysis. High-signal signified lesions and vessels within the lungs (or only vessels in control animals). [file Image_3.JPEG]

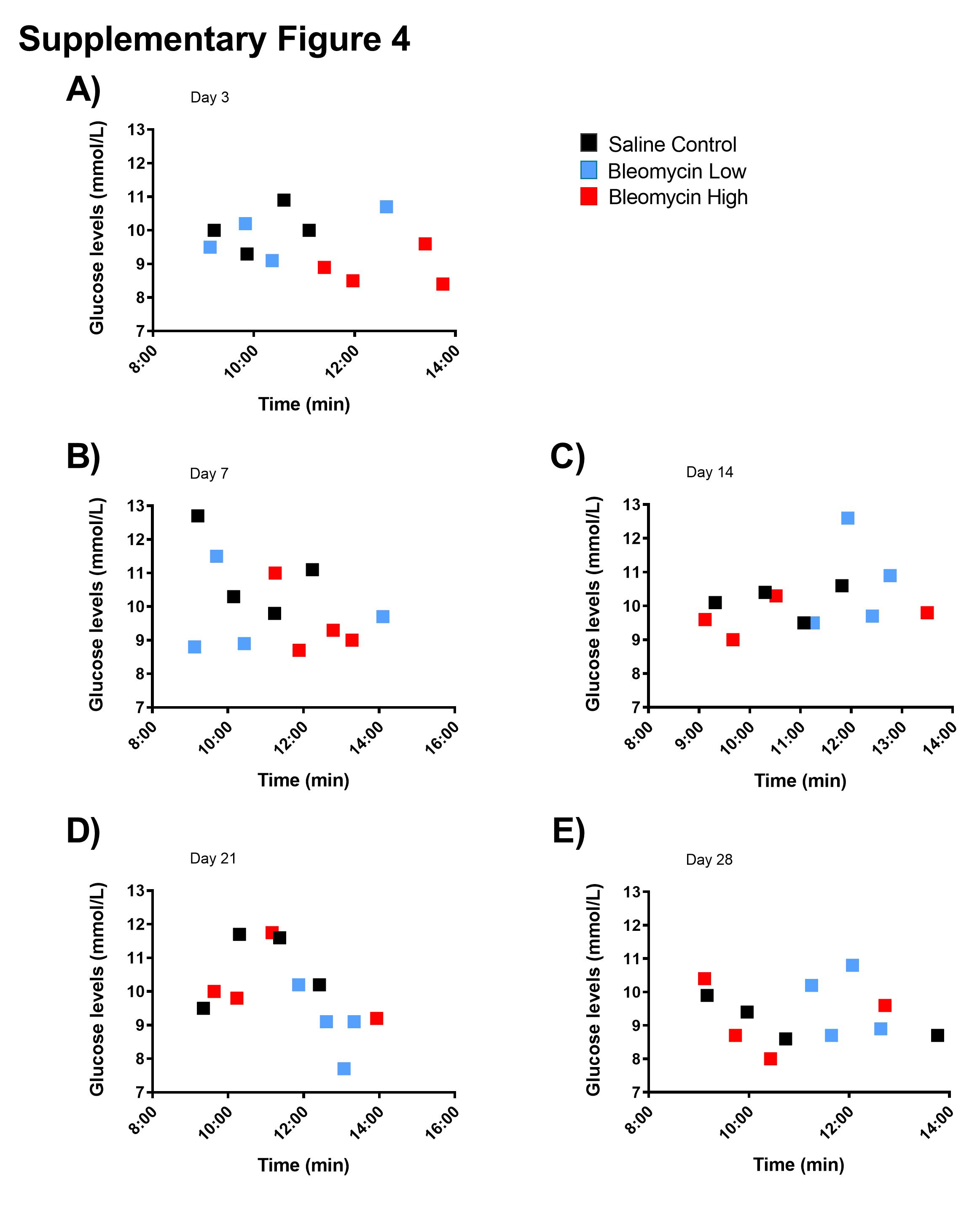

Supplement: FIGURE S4 — Glucose levels monitored before each imaging scan. Blood glucose measurements before each scan session from day 3 (A), 7 (B), 14 (C), 21 (D) and 28 (E). [file Image_4.JPEG]

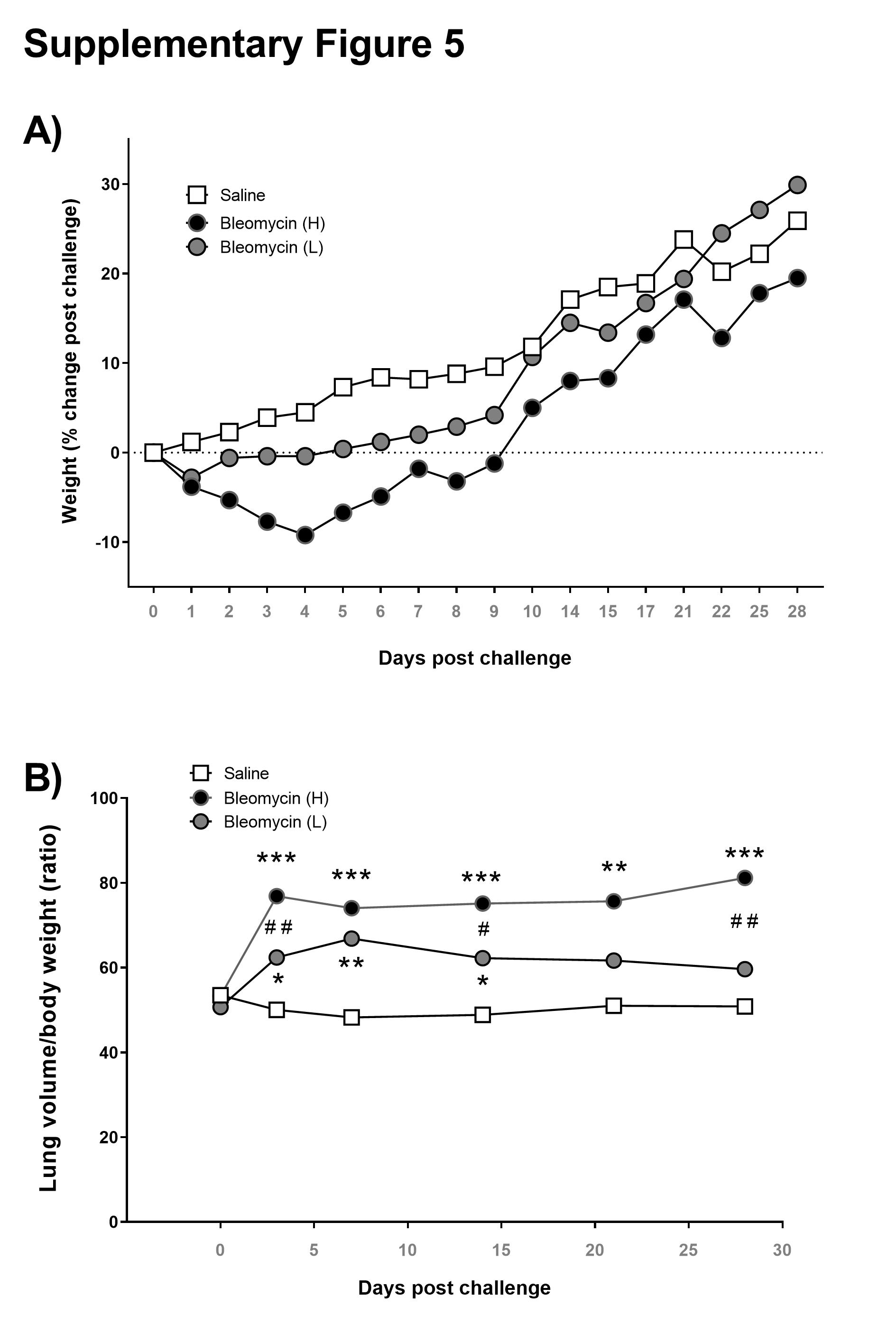

Supplement: FIGURE S5 — Body weight and disproportional lung volume increase in bleomycin-challenged rats. Body weight (A) and lung-ratio presented (B) according to the split bleomycin-challenged group into low- vs. high-responders to bleomycin-induced lung injury. Lung ratio was generated by calculating lung volume per body weight at that particular scan session, and presented as mean ratio for the whole group Significance was indicated by * when p < 0.05; p < 0.01 by ** and p < 0.001 by *** when comparing bleomycin towards the saline control from the same time point. The comparison of various time points between bleomycin-challenged groups is expressed as # when p < 0.05 and ## when p < 0.01. [file Image_5.JPEG]

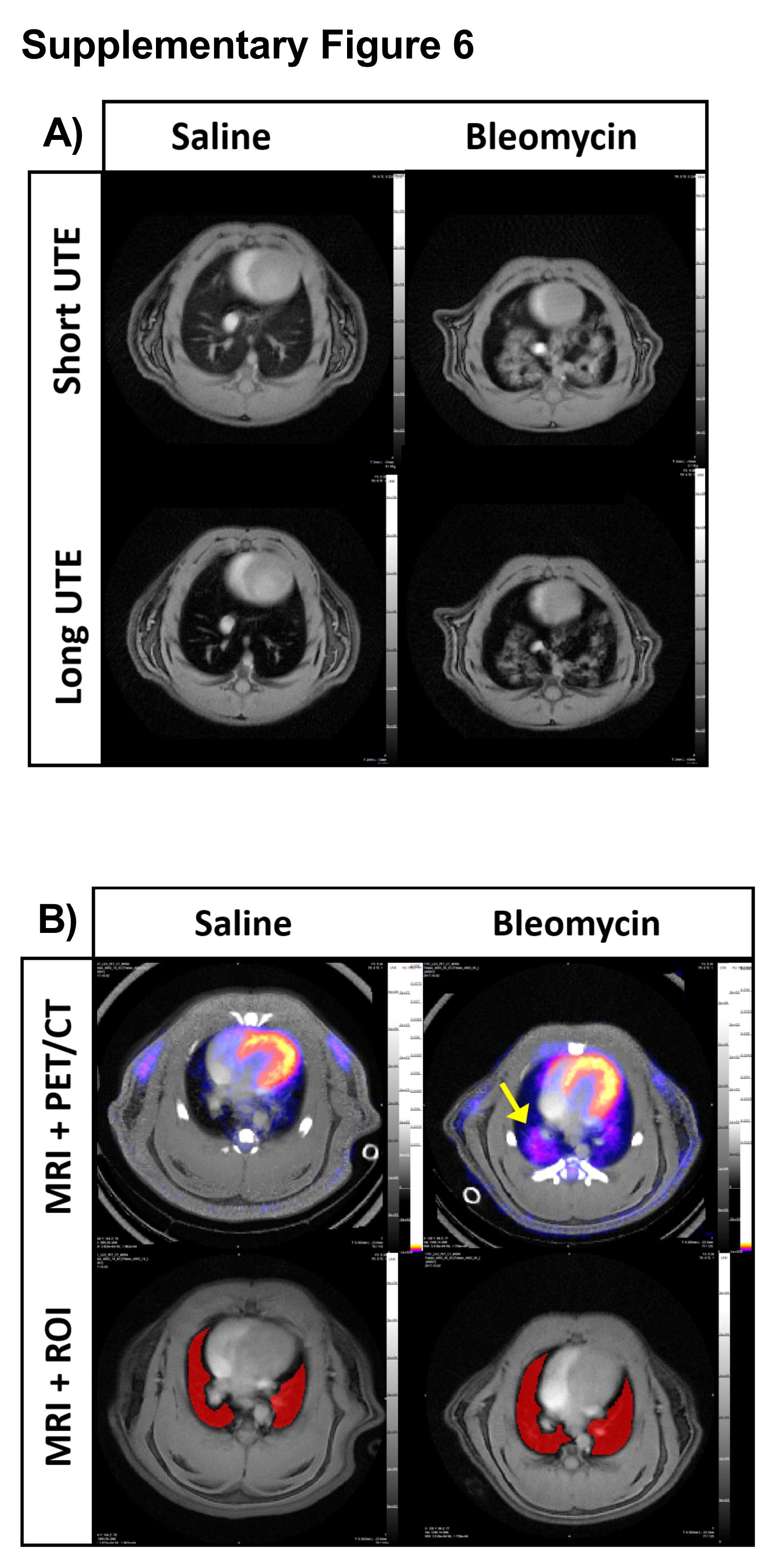

Supplement: FIGURE S6 — Representative images of MRI echo times and multimodality images overlapping. (A) two different scans using TELONG and TESHORT are presented in a bleomycin-challenged rat and one saline control rat. (B) Overlap images from both PET and MR, demonstrating how the different layers of images and ROI could be aligned. This method enables data extraction within the same ROI generated. At the same time images acquired by different techniques can be complementary when assessing lesions. [file Image_6.JPEG]
