# Supplementary material for: Imaging Biomarkers and Pathobiological Profiling in a Rat Model of Drug-Induced Interstitial Lung Disease Induced by Bleomycin
Source: Front Physiol. 2020 Jun 19;11:584. doi: 10.3389/fphys.2020.00584 (PMC7317035; doi:10.3389/fphys.2020.00584)
Supplement: DATA FILE S1 — Extended section of the Materials and Methods. Study severity and adverse effects were described according to the ARRIVE guidelines, as well as the power calculations and n-numbers. [file Table_1.DOCX]

**Supplementary data; Materials and Methods**

This study imposes burden to the animals through extensive imaging sessions but also the stimulus agent bleomycin used as a challenge. Therefore, additional supplementary section is included describing more in detail handling and criteria, according to the ARRIVE guidelines (Supplementary reference [1]).

*Adverse effects from bleomycin-challenge*

Bleomycin is a drug and challenging agent known to induced inflammation in the lungs during the first week when administrated as a single dose via the intratracheal (i.t.) route. Upon challenge with bleomycin, the rats were expected to lose weight. Therefore, special diet was added once the animals received bleomycin i.t. The food was both given as extruded feed as well as mashed food (adding tap water to be able to mash it up) and introduced at the bottom of the cage for easier access and intake.

*Termination endpoints upon weight loss*

According to the ethical permit, the rats were also monitored on a daily basis during the first week post challenge. Termination endpoints were taken if the animals lost more than 15% of the initial body weight. If animals were starting to lose close to 15 % of their baseline weight, the animal was removed from the study and terminated (which constitutes a termination criteria according to ethical permit). If the weight started to increase within this observation period, the animal was continued allowed to be part the study.

*Adverse effects from the imaging sessions*

Any type of imaging in live animals demand that the animals need to be sedated and put under anaesthesia during long periods of time (, approximately 1.5 h according to our workflow during each scan session). Anastasia might induce nausea and drowsiness, which normally ceases within minutes to an hour after awakening. To minimise this, the imaging sessions were planned and optimised in order to sample imaging data as fast as possible and combine the modalities in such order to enhance efficiency.

*Animals omitted from the study*

From the imaging group (n=4 saline controls and n=8 bleomycin challenged rats), all animals that entered the study also completed the study until planned termination day (day 28) directly after the last live imaging session. From the non-imaging group, 42 animals entered the study, while 33 were included in total (n=10 saline controls and n=23 bleomycin challenged rats). The 9 animals that were removed from the study were all bleomycin-challenged rats that were not able to sustain the bodyweight or keep up with criteria due to severe illness, thus those animals were terminated.

Data generated from the 9 rats that were excluded from the study, were not part of the data analysis, thus excluded completely from the study cohort.

*Randomisation and study plan*

During study set-up, all rats were divided in groups firstly, then challenged with bleomycin or saline as control. Initial study design included a minimum of two saline controls at each termination time-point in both the non-imaging group as well as within the imaging groups. Then, each group of rats was divided into 4-6 rats.

Due to loss of animals, particularly at later time points some groups were reduced to 3 rats. This was however enough to be able to compare various time points while at the same time did not require a large amount of animals as starting material, being able to keep the animal numbers optimal. Assumption that 20-25% of the bleomycin-challenged animals would be removed from the study due to humane endpoints, a simple formulation was applied to check for power (Supplementary reference [2]):

“ Corrected sample size = Sample size/ (1− [% attrition/100]) ”

In total: 40 rats were challenged with bleomycin, and 14 rats were controls.

Imaging group: n=4 Saline and n=8 bleomycin

Non-imaging group: n=10 Saline and n=32 bleomycin (while only 23 survived).

From the initial 40 rats that received bleomycin (corrected sample size), 9 were removed during the study, resulting in 31 bleomycin-challenged rats at the end of the study (actual sample size).

- Corrected sample size = 40
- Sample size = 31
- % attrition= 22.5% (between 20%-25%)

Final calculation: 40 = 31/(1− [22.5/100])

References:

1. Kilkenny, C., et al., *Animal research: reporting in vivo experiments: the ARRIVE guidelines.* Br J Pharmacol, 2010. **160**(7): p. 1577-9.

2. Charan, J. and N.D. Kantharia, *How to calculate sample size in animal studies?* J Pharmacol Pharmacother, 2013. **4**(4): p. 303-6.
