# Supplementary material for: Imaging Biomarkers and Pathobiological Profiling in a Rat Model of Drug-Induced Interstitial Lung Disease Induced by Bleomycin
Source: Front Physiol. 2020 Jun 19;11:584. doi: 10.3389/fphys.2020.00584 (PMC7317035; doi:10.3389/fphys.2020.00584)
Supplement: DATA FILE S2 — Supporting material of the total lung volume from a pilot study. The total lung volume increase was observed in another experiment and set of animals, indicating the robustness of this imaging biomarker. [file Table_2.DOCX]

*Figure A: Total lung volume at 12 and 28 days post bleomycin i.t. instillation. The significantly increased lung volume in the bleomycin group is evident, showing the similar pattern as in the main large study presented. Also in here, in the small study, seems to be a separation of two groups among all rats that received bleomycin, as presented in Figure B.*

*Figure B: Separating the bleomycin group into high- vs. low responders according to the separation at day 28 seen in Figure A. Once separated into two distinguished bleomycin groups (according to high- vs. low responders), there is a higher significant difference between saline and both bleomycin groups, as well as significant difference between the high vs. low responder group, at day 28 post bleomycin challenge.*

*Animals: n=12 in total*

*Bodyweight at day 0 of the study: 260g (±5g).*

*Groups: Saline n=2; Bleomycin n=10*

*Imaging: MRI*

*Readout: Total lung volume (mm^3^)*

*Challenge: Saline or Bleomycin i.t. instillation (1000 iU given to all rats) (Sigma Aldrich, St. Louis, USA), in a total volume of 200µl.*

*All animal handling and procedures considering the i.t. challenge, during imaging sessions and anesthesia, as well as data processing from MRI readout was performed in exactly the same way as for the main larger study.*

*This additional small study was done to confirm the imaging biomarker of pathological “lung volume increase”, upon bleomycin challenge. Also here, we observed the phenomenon of separation between the bleomycin-challenged rats at day 28.*
